# Evaluation of the Anticancer Potential of Compound 107 via the VEGFA and VEGFR2 (*KDR*) Signaling Pathway in Colorectal Cancer: Integrative Analysis of Clinical, In Silico, and In Vitro Approaches

**DOI:** 10.3390/ijms27219621

**Published:** 2026-10-27

**Authors:** Ayca Tas, Cemile Zontul, Gökhan Dogan, Hakki Coskun, Husnu Cagri Genc, Kenan Goren, Tugba Agbektas, Veysel Tahiroglu, Alakbar Huseynzada, Ulviyya Hasanova, Gonca Kabak, Zeynep Deniz Sahin Inan, Hatice Ozer, Yavuz Silig

**Affiliations:** 1 Department of Medical Biochemistry, Faculty of Medicine, Sivas Cumhuriyet University, 58140 Sivas, Türkiye; aycatas@cumhuriyet.edu.tr (A.T.); drgokhandogan13@gmail.com (G.D.); gonca.kabak24@gmail.com (G.K.); 2 Department of Chemistry and Chemical Prossesing Technologies, Yıldızeli Vocational School, Sivas Cumhuriyet University, 58140 Sivas, Türkiye; cemilezontul@cumhuriyet.edu.tr; 3 Department of General Surgery, Faculty of Medicine, Sivas Cumhuriyet University, 58140 Sivas, Türkiye; hacoskun@cumhuriyet.edu.tr (H.C.); cagrigenc@cumhuriyet.edu.tr (H.C.G.); 4 Department of Petroleum and Natural Gas Engineering, Faculty of Engineering, Şırnak University, 73000 Şırnak, Türkiye; kenangoren49@gmail.com; 5 Department of Food Processing Technologies Services, Yıldızeli Vocational School, Sivas Cumhuriyet University, 58140 Sivas, Türkiye; tubaagbektas@cumhuriyet.edu.tr; 6 Department of Nursing, Faculty of Health Sciences, Şırnak University, 73000 Şırnak, Türkiye; veysel.tahiroglu@sirnak.edu.tr; 7 Industrial Chemistry Research Laboratory (ICRL), Baku State University, Z. Khalilov 33, Baku AZ 1148, Azerbaijan; alakbar.huseynzada1117@gmail.com; 8 Islamic World Educational, Scientific and Cultural Organization (ICESCO), Biomedical Materials Department, Baku State University, Z. Khalilov 33, Baku AZ 1148, Azerbaijan; ulviyyahasanova12@gmail.com; 9 Department of Histology and Embryology, Faculty of Medicine, Sivas Cumhuriyet University, 58140 Sivas, Türkiye; zinan@cumhuriyet.edu.tr; 10 Department of Medical Pathology, Faculty of Medicine, Sivas Cumhuriyet University, 58140 Sivas, Türkiye; hozer@cumhuriyet.edu.tr

**Keywords:** colorectal cancer, Compound 107, VEGFA, VEGFR2/*KDR*, angiogenesis, apoptosis, molecular docking

## Abstract

The VEGFA and VEGFR2/*KDR* signaling pathway is a major regulator of tumor angiogenesis and colorectal cancer (CRC) progression. This study investigated the anticancer effects of Compound **107** using integrated clinical, in vitro, and in silico approaches. VEGFA and KDR expression was evaluated in paired colorectal tumor and adjacent non-neoplastic tissues, while molecular docking and ADMET analyses were performed to characterize Compound **107**. Its effects on viability, apoptosis, cell-cycle distribution, *VEGFA/KDR* gene expression, and VEGFA and VEGFR2 protein levels were assessed in HT-29 cells. *VEGFA* and *KDR* expression was significantly increased in CRC tissues by 1.98- and 2.02-fold, respectively (both *p* < 0.001), accompanied by increased protein immunoreactivity (both *p* < 0.0001). Compound **107** showed favorable predicted binding to VEGFA (−8.60 kcal/mol) and VEGFR2 (−8.30 kcal/mol), although ADMET analysis indicated limitations related to pharmacokinetic and toxicity profiles. Compound **107** reduced MTT-based metabolic viability in a concentration- and time-dependent manner, increased early apoptosis, decreased the G0/G1 population, and increased the S-phase population. Moreover, *VEGFA* and *KDR* gene expression and corresponding protein fluorescence intensities were significantly reduced following treatment. Collectively, Compound **107** reduced metabolic viability and increased early apoptosis, accompanied by reduced VEGFA and KDR expression in HT-29 cells. These findings indicate an association between the cellular effects of Compound **107** and changes in VEGFA/VEGFR2-related molecular markers; however, they do not demonstrate direct inhibition of the VEGFA/VEGFR2 signaling pathway. Further mechanistic studies are required to clarify this relationship.

## 1. Introduction

Colorectal cancer (CRC) is a complex malignancy characterized by cell proliferation, invasion, metastasis, and alterations in the tumor microenvironment. Neovascularization is critically important for the growth and metastatic spread of solid tumors, and one of the key regulators of this process is the vascular endothelial growth factor (VEGF) family. Current multi-omics analyses have shown that members of the VEGF family (including VEGFA) are increased in colon adenocarcinoma tissues and that the VEGF family is closely related to CRC biology [1]. Vascular endothelial growth factor A (VEGFA), a key member of the VEGF family, plays a central role in regulating tumor angiogenesis. In colorectal cancer, it has been shown that long non-coding RNA 01503 (LINC01503) regulates VEGFA expression via microRNA-342-3p (miR-342-3p) and heat shock protein 60 (HSP60), and that increased VEGFA promotes endothelial cell migration and tube formation [2]. The biological effects of VEGFA occur largely through the vascular endothelial growth factor receptor 2 (VEGFR2) encoded by *KDR*. Demonstration of VEGFR2 expression in immune cells in the tumor microenvironment of patients with colorectal cancer suggests that this receptor may contribute to the regulation of the tumor microenvironment in addition to angiogenesis [3]. Single-cell ribonucleic acid (RNA) sequencing studies have revealed the importance of the VEGFA and VEGFR2 pathway in CRC. It has been reported that VEGFR2 is particularly enriched in angiogenic endothelial cells in CRC tissues and that the VEGFA and VEGFR2 endothelial cell-specific molecule 1 (ESM1) signaling pathway supports angiogenesis as well as the proliferation and migration of tumor cells [4]. Furthermore, the long non-coding RNA 01106 (LINC01106)/microRNA-449b-5p (miR-449b-5p) and VEGFA signaling pathway, regulated by YTH N6-methyladenosine RNA-binding protein 1 (YTHDF1), has been shown to play a role in CRC vascularization, and VEGFA has been shown to be a common target of different molecular regulatory mechanisms [5]. VEGFA signaling is also closely related to intracellular proliferation and survival. Phosphatase and tensin homolog/protein kinase B (PTEN/AKT)-mediated sialylation of ubiquitin-specific peptidase 13 (USP13) suppresses VEGFA expression and angiogenesis in CRC [6]. Similarly, it has been shown that suppression of hypoxia-inducible factor-1 alpha (HIF-1α) in hypoxic colon cancer cells reduces VEGF and VEGFR2 levels, thereby inhibiting Wnt/β-catenin signaling, proliferation, and migration [7]. When these findings are considered together, it appears that the HIF-1α/VEGF–VEGFR2, phosphoinositide 3-kinase/protein kinase B (PI3K/AKT), and Wnt/β-catenin pathways are interrelated in regulating the angiogenic and proliferative properties of colon cancer.

Although targeting the VEGF/VEGFR signaling pathway is an important approach in the treatment of advanced CRC, the variability in response to anti-angiogenic therapies among patients and the limited availability of reliable biomarkers that can predict treatment response pose a significant problem [8]. Furthermore, the heterogeneity of tumor vessels and the resistance mechanisms that develop against anti-angiogenic therapies highlight the need to investigate novel molecules that can affect VEGF/VEGFR signaling [9]. In this context, Compound **107**, evaluated in our study, is a synthetic compound with –CH=N– bonds of azomethine/Schiff base character. The synthesis of Compound **107** was performed based on a previously reported method for compounds containing an azomethine group. It has been previously shown that Synthetic compounds containing an azomethine group can reduce the viability of cancer cells and alter the expression of genes associated with wingless type MMTV integration site/mitogen-activated protein kinase (Wnt/MAPK) signaling patterns [10]. Therefore, evaluating the effect of Compound **107** on colorectal cancer cells via the angiogenesis-related VEGFA and VEGFR2 pathways is important for elucidating the possible anticancer mechanisms of this compound. The possible pharmacological relationship between the azomethine structure and VEGFR2 also supports this hypothesis. It has been reported that some newly synthesized Schiff derivatives can exhibit antiproliferative activity and some derivatives can inhibit VEGFR2 kinase activity [11]. However, this finding does not indicate that Compound **107** is a direct VEGFR2 inhibitor; it provides a scientific justification for experimentally investigating the possible effect of Compound **107** on the VEGFA and VEGFR2 signaling pathway. The identification of VEGFA as an important molecular target associated with CRC progression in current multi-omics and single-cell analyses further supports the importance of this axis in the evaluation of new therapeutic candidates [12].

Recent advances in structure-based drug discovery have highlighted the importance of molecular docking and molecular dynamics simulations in identifying and characterizing potential angiogenesis-targeting compounds. Recent studies have investigated the interactions of small molecules with VEGFA, providing structural insights into ligand recognition, binding conformations, and the molecular determinants of protein–ligand interactions. In particular, computational investigations of anti-VEGF agents have demonstrated the utility of integrating molecular docking with binding affinity prediction and structural modeling to characterize VEGFA–ligand interactions [13,14]. Furthermore, recent structure-based drug design studies have focused on the development of novel VEGFR2-targeting compounds for colorectal cancer. The integration of molecular docking, molecular dynamics simulations, and experimental validation has been employed to investigate the binding characteristics and biological effects of novel VEGFR2 inhibitors in colorectal cancer models, including HT-29 cells [15,16]. These findings highlight the relevance of structure-based approaches in the preliminary assessment of compounds targeting angiogenesis-related molecular mechanisms. Accordingly, evaluating the predicted interactions of Compound **107** with VEGFA and VEGFR2, alongside its effects on gene and protein expression, may provide complementary insights into its potential role in modulating angiogenesis-associated signaling in colorectal cancer.

Despite the established involvement of the VEGFA/VEGFR2 axis in colorectal cancer, the potential effects of Compound **107** on this angiogenesis-associated signaling pathway remain unexplored. In particular, it is unknown whether Compound **107** is associated with changes in VEGFA and VEGFR2 expression in colorectal cancer cells and whether its predicted interactions with these proteins provide a plausible structural basis for further investigation of this relationship. Therefore, the central hypothesis of the present study was that Compound **107** may exert anticancer effects in HT-29 colorectal cancer cells that are associated with modulation of the VEGFA/VEGFR2 axis, while molecular docking may provide complementary structural hypotheses regarding its possible interactions with VEGFA and VEGFR2. To address this hypothesis, the biological effects of Compound **107** were investigated experimentally at the gene and protein expression levels, while molecular docking was used to explore its predicted interactions with VEGFA and VEGFR2. The docking results were interpreted as hypothesis-generating computational evidence and were not considered proof of direct target inhibition or experimentally determined binding affinity.

## 2. Results

### 2.1. Demographic Analysis of Patients

A total of 32 patients were included in the study. The mean age was 64.78 ± 9.73 years. Of the patients, 21 (65.6%) were male and 11 (34.4%) were female. The most common tumor localization was the rectosigmoid colon (37.5%), and most tumors were classified as Grade 2 (65.6%). Neoadjuvant therapy had been administered to two patients (6.3%). The demographic, clinical, and clinicopathological characteristics of the patients are presented in Table 1.

### 2.2. Tissue Expression of VEGFA and KDR

**Figure 1:**
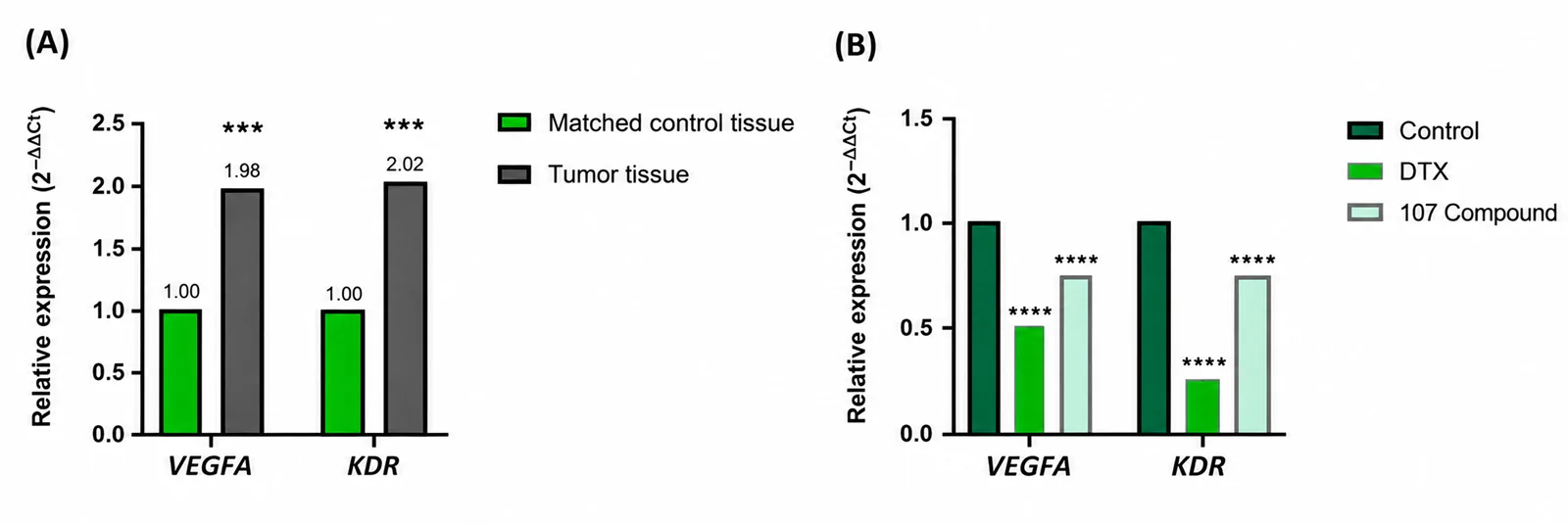
Relative expression levels of *VEGFA* and *KDR* in tissue samples and HT29 cell groups. (**A**) Relative expression of *VEGFA* and *KDR* in tumor tissues compared with matched tumor-adjacent non-neoplastic tissues. Relative expression was calculated using the 2^−ΔΔCt^ method, with matched tumor-adjacent non-neoplastic tissues normalized to 1. Statistical comparisons were performed on paired ΔCt values using the paired-samples *t*-test. *** *p* < 0.001 versus matched tumor-adjacent non-neoplastic tissues. (**B**) Relative expression of *VEGFA* and *KDR* in control, DTX-treated, and Compound **107**-treated HT29 cells. Statistical comparisons were performed on ΔCt values using two-way ANOVA followed by Dunnett’s multiple-comparisons test, with the control group used as the reference. **** *p* < 0.0001 versus control.

Tumor tissue VEGFA ΔCt values were significantly lower than those measured in matched tumor-adjacent non-neoplastic tissues (5.80 ± 2.88 vs. 6.78 ± 2.75). The mean paired ΔΔCt was −0.98 (95% CI: −1.14 to −0.83), and the difference was statistically significant, t(31) = −13.21, *p* < 0.001. Based on the 2^−mean ΔΔCt^ method, VEGFA expression was 1.98-fold higher in tumor tissue. Similarly, *KDR* ΔCt values were significantly lower in tumor tissues than in matched tumor-adjacent non-neoplastic tissues (11.30 ± 2.48 vs. 12.31 ± 2.30). The mean paired ΔΔCt was −1.01 (95% CI: −1.20 to −0.82), and the paired difference was statistically significant, t(31) = −10.84, *p* < 0.001. This corresponded to a 2.02-fold increase in *KDR* expression in tumor tissue. The paired tissue comparisons are summarized in Table 2 and illustrated in Figure 1A.

### 2.3. Associations with Histological and Clinical Characteristics

Neither *VEGFA* nor *KDR* tumor-to-control ΔCt differences varied significantly across histological grade groups. The Kruskal–Wallis test showed no significant difference for *VEGFA*, H(2) = 0.760, *p* = 0.684, or *KDR*, H(2) = 3.130, *p* = 0.209 (Table 3).

Because the Grade 3 group included only two patients, findings related to this subgroup should be interpreted cautiously. No significant differences in *VEGFA* or *KDR* ΔΔCt values were observed according to neoadjuvant treatment status (Mann–Whitney U test: *VEGFA*, U = 9.00, exact *p* = 0.121; *KDR*, U = 10.50, exact *p* = 0.145). Similarly, tumor localization was not significantly associated with *VEGFA*, H(9) = 4.165, *p* = 0.900, or *KDR*, H(9) = 5.421, *p* = 0.796, ΔΔCt values. Exploratory subgroup analyses further demonstrated that *VEGFA* and *KDR* tumor-to-control ΔCt differences were not significantly associated with diabetes mellitus, hyperlipidemia, hypertension, cardiovascular disease, history of cancer, alcohol consumption, smoking status, or dietary pattern, with all *p* > 0.05. These findings should be interpreted cautiously because of the small and uneven subgroup sizes.

### 2.4. Correlation Between VEGFA and KDR

A statistically significant positive correlation was observed between *VEGFA* and *KDR* tumor-to-control ΔCt differences (Spearman’s ρ = 0.358, *p* = 0.044, n = 32), indicating that changes in the expression of the two genes tended to occur in the same direction (Table 4).

### 2.5. Histopathological Findings

**Figure 2:**
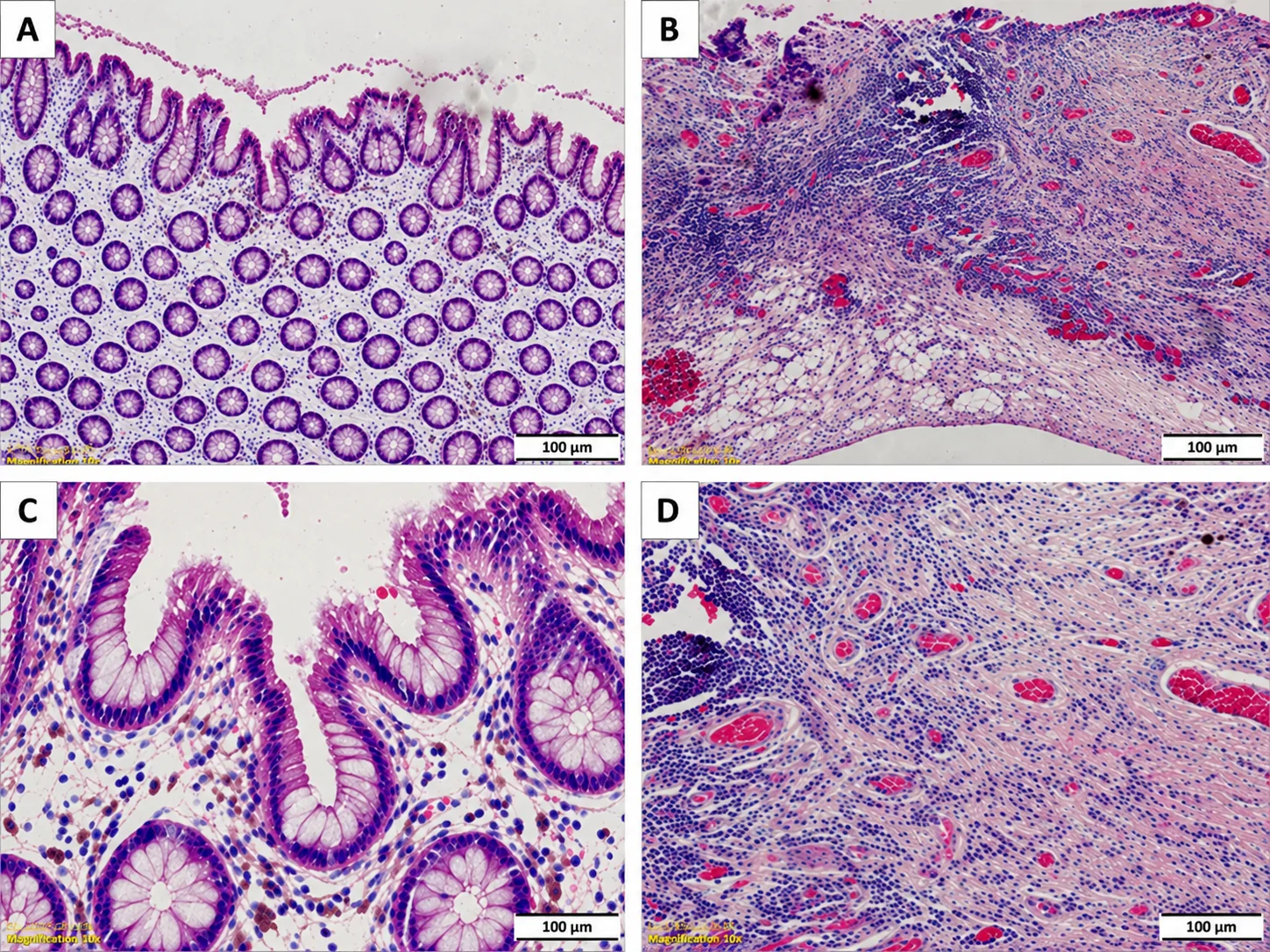
Representative H&E-stained sections of tumor-adjacent non-neoplastic colorectal tissue and colorectal adenocarcinoma tissue. (**A**) Tumor-adjacent non-neoplastic colorectal tissue exhibiting preserved mucosal architecture and regularly arranged tubular crypts (×10 objective; scale bar = 100 μm). (**B**) Colorectal adenocarcinoma tissue displaying disruption of the glandular architecture, infiltrative tumor growth, desmoplastic stromal changes, and inflammatory cell infiltration (×10 objective; scale bar = 100 μm). (**C**) Higher-magnification view of tumor-adjacent non-neoplastic colorectal mucosa showing an intact simple columnar epithelium, basally located nuclei, and abundant goblet cells (×40 objective; scale bar = 20 μm). (**D**) Higher-magnification view of colorectal adenocarcinoma tissue demonstrating tumor-associated desmoplastic stroma, numerous vascular profiles, and inflammatory cell infiltration (×20 objective; scale bar = 50 μm).

Microscopic examination of H&E-stained sections revealed pronounced morphological differences between tumor-adjacent non-neoplastic colorectal tissues and colorectal adenocarcinoma tissues (Figure 2). The tumor-adjacent non-neoplastic tissues displayed well-preserved mucosal architecture, characterized by regularly arranged tubular crypts lined with simple columnar epithelial cells containing basally located nuclei and numerous goblet cells. The surface and crypt epithelium remained intact, with no evident architectural distortion, cellular atypia, dysplastic changes, or neoplastic alterations. In contrast, colorectal adenocarcinoma tissues exhibited extensive disruption and loss of the normal glandular architecture. The neoplastic glands were irregular in size, shape, and distribution; they were frequently fused and demonstrated infiltrative growth into the surrounding stroma. The tumor cells displayed enlarged, hyperchromatic, and pleomorphic nuclei, accompanied by loss of normal cellular polarity and increased mitotic activity. Prominent desmoplastic stromal changes, numerous vascular profiles, focal inflammatory cell infiltration, and scattered areas of necrosis were also observed within the tumor tissues. Collectively, these morphological findings were consistent with the characteristic histopathological features of colorectal adenocarcinoma and clearly distinguished the tumor tissues from the corresponding tumor-adjacent non-neoplastic colorectal mucosa.

### 2.6. Immunohistochemical Findings

**Figure 3:**
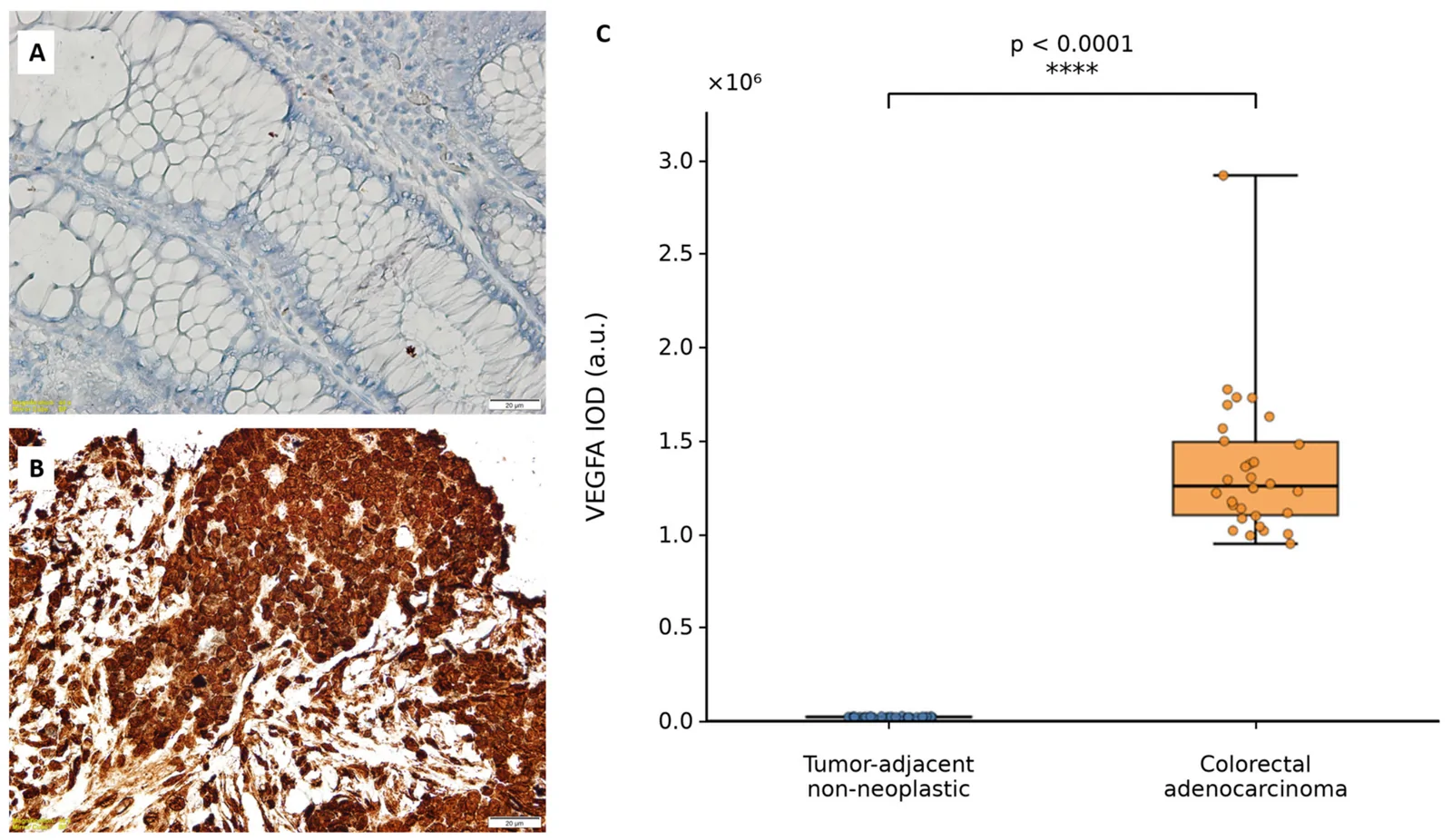
Immunohistochemical expression of VEGFA in tumor-adjacent non-neoplastic colorectal tissue and colorectal adenocarcinoma tissue. (**A**) Representative tumor-adjacent non-neoplastic colorectal tissue showing absent to weak VEGFA immunoreactivity in the colonic epithelium. (**B**) Representative colorectal adenocarcinoma tissue exhibiting strong and diffuse cytoplasmic VEGFA immunoreactivity within neoplastic epithelial cells. (**C**) Semi-quantitative ImageJ analysis of VEGFA immunoreactivity based on integrated optical density (IOD). VEGFA IOD values were significantly higher in colorectal adenocarcinoma tissues than in the corresponding paired tumor-adjacent non-neoplastic tissues. Each dot represents the IOD measurement obtained from one tissue specimen; a total of 32 paired cases were analyzed. Box plots indicate the median and interquartile range, while the whiskers represent the minimum and maximum values. Statistical significance was determined using the Wilcoxon signed-rank test (**** *p* < 0.0001). Scale bars = 20 μm.

**Figure 4:**
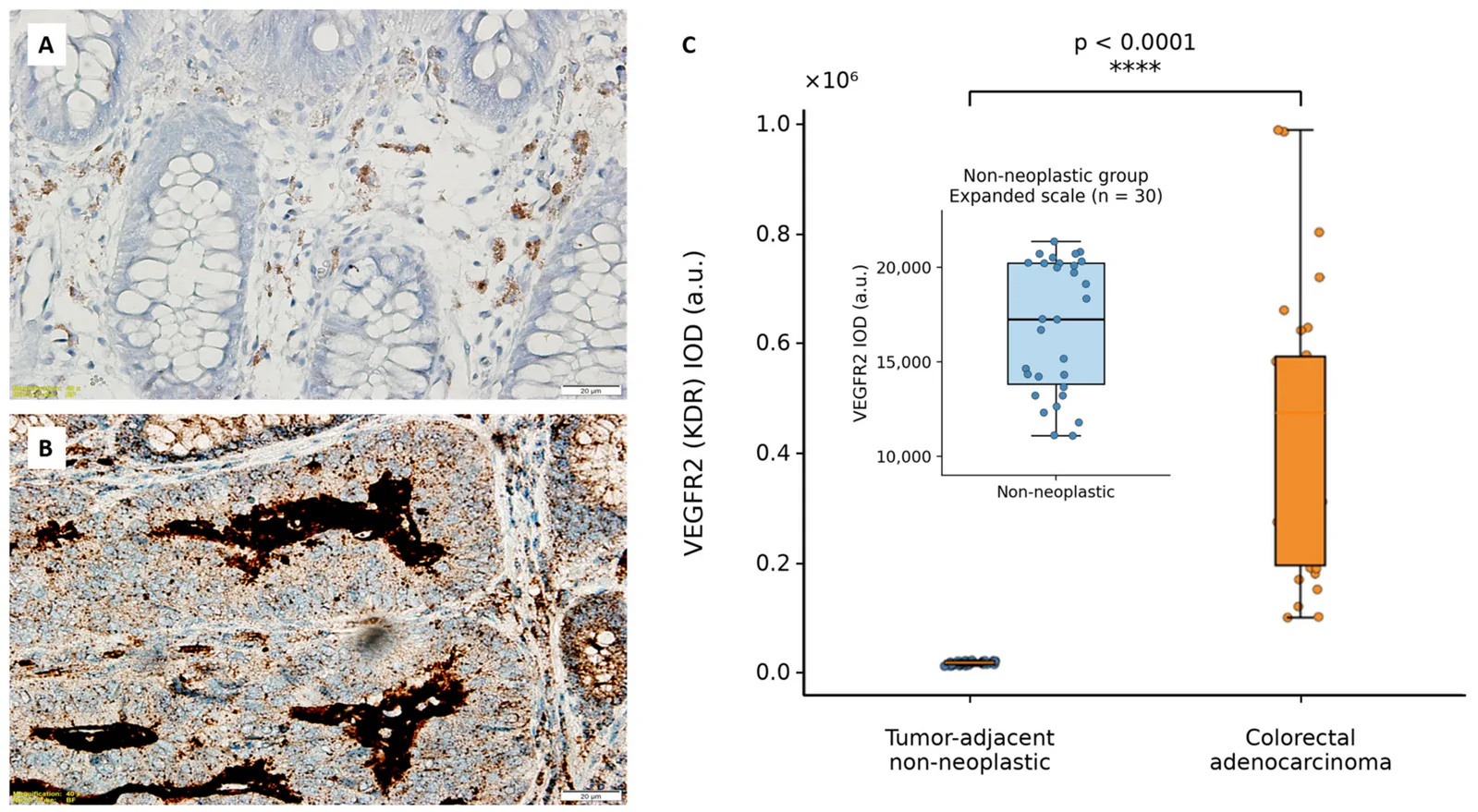
Immunohistochemical expression of VEGFR2 in tumor-adjacent non-neoplastic colorectal tissue and colorectal adenocarcinoma tissue. (**A**) Representative tumor-adjacent non-neoplastic colorectal tissue showing weak and focal VEGFR2 immunoreactivity. (**B**) Representative colorectal adenocarcinoma tissue exhibiting markedly increased VEGFR2 immunoreactivity within neoplastic glandular structures and tumor-associated stromal elements. (**C**) Semi-quantitative ImageJ analysis of VEGFR2 immunoreactivity based on integrated optical density (IOD). VEGFR2 IOD values were significantly higher in colorectal adenocarcinoma tissues than in the corresponding paired tumor-adjacent non-neoplastic tissues. Each dot represents the IOD measurement obtained from one tissue specimen; a total of 32 paired cases were analyzed. Box plots indicate the median and interquartile range, while the whiskers represent the minimum and maximum values. The inset displays the same 32 tumor-adjacent non-neoplastic measurements on an expanded linear scale to visualize their variability. Statistical significance was determined using the Wilcoxon signed-rank test (**** *p* < 0.0001). Scale bars in panels (**A**,**B**) = 20 μm.

Immunohistochemical analysis revealed markedly increased VEGFA and VEGFR2 immunoreactivity in colorectal adenocarcinoma tissues compared with the corresponding tumor-adjacent non-neoplastic colorectal tissues (Figure 3 and Figure 4). In tumor-adjacent non-neoplastic colorectal tissues, VEGFA immunoreactivity was absent to weak in the colonic epithelium, with focal staining observed only in scattered epithelial and stromal cells. In contrast, colorectal adenocarcinoma tissues exhibited strong and diffuse VEGFA immunoreactivity, predominantly localized within the cytoplasm of neoplastic epithelial cells. Quantitative ImageJ version 1.54u8 analysis confirmed that VEGFA integrated optical density (IOD) values were significantly higher in colorectal adenocarcinoma tissues than in the paired tumor-adjacent non-neoplastic tissues (Wilcoxon signed-rank test, *p* < 0.0001; Figure 3C). VEGFR2 immunoreactivity was weak and focal in tumor-adjacent non-neoplastic colorectal tissues and was primarily detected in scattered stromal and vascular cells, with minimal staining in the epithelial compartment. In contrast, colorectal adenocarcinoma tissues demonstrated markedly increased VEGFR2 immunoreactivity within neoplastic glandular structures and the tumor-associated stromal and vascular compartments. Quantitative ImageJ analysis showed that VEGFR2 IOD values were significantly higher in colorectal adenocarcinoma tissues than in the corresponding paired tumor-adjacent non-neoplastic tissues (Wilcoxon signed-rank test, *p* < 0.0001; Figure 3C). Collectively, these findings demonstrated markedly increased VEGFA and VEGFR2 immunoreactivity in colorectal adenocarcinoma tissues relative to matched tumor-adjacent non-neoplastic colorectal tissues. Cellular localization was assessed descriptively on morphological grounds, whereas IOD measurements were obtained at the field level without separate quantification of tumor epithelial, endothelial, stromal, or immune-cell compartments. Therefore, these measurements indicate increased immunoreactivity but do not establish cell-type-specific expression or activation of the VEGFA/VEGFR2 signaling pathway.

### 2.7. İn Silico ADMET and Drug-Likeness Results

In pharmaceutical research, the ADMET (Absorption, Distribution, Metabolism, Elimination, and Toxicity) profile is one of the most critical elements in determining the efficacy and safety of drug candidates. It comprehensively describes the behavior of a compound in biological systems [17]. ADMET properties not only determine a compound’s pharmacokinetic properties but also directly influence its pharmacodynamic effects. Therefore, ADMET analysis is an essential step in the drug development process for screening or optimizing potential drug candidates [18]. In this study, the ADMET profiles of the synthesized compound **107** were comprehensively evaluated using the online in silico prediction platform ADMETlab 2.0 [19]. It is crucial to emphasize that the obtained data represent computationally predicted probabilities and mathematical categorizations, and they should not be interpreted as absolute, experimentally measured pharmacokinetic parameters. Nevertheless, these computational predictions provide preliminary, hypothesis-generating insights into the potential pharmacokinetic and pharmacodynamic characteristics of the compound prior to experimental validation (Table 5).

The molecular weight of compound **107** (593.19 g/mol) exceeds the threshold proposed by Lipinski’s rule of five (<500 g/mol), suggesting that the compound may be associated with reduced oral bioavailability. Similarly, the number of hydrogen bond acceptors (nHA = 16) is higher than the recommended value (<12), while the number of hydrogen bond donors (nHD = 3) remains within the acceptable range. Furthermore, the compound possesses a high topological polar surface area (TPSA = 230.43 Å^2^), which is considerably above the ideal limit of 140 Å^2^ and generally may be associated with reduced membrane permeability and intestinal absorption. The calculated logP value of 2.82 falls within the optimal range (0–5), indicating a balanced lipophilic profile that suggesting a relatively balanced lipophilic profile that may influence aqueous solubility and membrane interactions. Nevertheless, the predicted human intestinal absorption probability (HIA = 0.00) and the Caco-2 permeability value (−5.387) suggest potentially low gastrointestinal absorption and limited passive diffusion across epithelial membranes. These findings are in agreement with the elevated molecular weight and high polar surface area of the compound. The probability of blood–brain barrier penetration was predicted to be negligible (BBB = 0.00), indicating that compound **107** had a low predicted probability of central nervous system penetration. This prediction may potentially be associated with a lower likelihood of CNS exposure; however, its biological relevance requires experimental confirmation. The plasma protein binding value (PPB = 82.026%) is below the critical threshold of 90%, suggesting a relatively moderate predicted degree of plasma protein binding; however, the actual free fraction in systemic circulation requires experimental determination. In addition, the predicted volume of distribution (VD = 0.057 L/kg) falls within the optimal physiological range (0.04–20 L/kg), suggesting a predicted distribution volume within the reported physiological range; however, this does not necessarily establish adequate tissue distribution in vivo. From a metabolic perspective, compound **107** exhibited very low probabilities of inhibiting CYP2D6 (0.033) and CYP3A4 (0.013), suggesting a relatively low predicted probability of inhibition of these cytochrome P450 enzymes and, consequently, a potentially lower likelihood of CYP-mediated drug–drug interactions. The predicted elimination half-life (T_1/2_ = 1.081 h) is relatively short; however, the actual elimination kinetics of the compound should be established experimentally. The toxicity predictions revealed several unfavorable characteristics. The predicted probability of hERG channel blockade (0.796) suggests a potentially elevated risk that warrants further experimental evaluation; however, this prediction alone cannot establish cardiotoxicity or QT interval prolongation. Likewise, the predicted human hepatotoxicity value (H-HT = 0.81) suggests a relatively high predicted probability of hepatotoxicity, which should be considered a potential safety signal requiring experimental validation. Moreover, the AMES toxicity prediction (0.999) indicates a high probability of a positive Ames test response and therefore represents a potential mutagenicity-related safety signal that requires experimental confirmation, while the rat oral acute toxicity probability (0.794) classifies the compound as having a relatively high probability of acute oral toxicity in the rat model. Overall, the in silico ADMET analysis suggests that compound **107** possesses a moderate predicted lipophilic character and relatively low predicted probabilities of CYP2D6 and CYP3A4 inhibition. However, its high molecular weight, elevated polarity, low predicted gastrointestinal absorption, and several unfavorable toxicity-related predictions represent potential pharmacokinetic and safety concerns. Importantly, these findings are based exclusively on computational predictions and should not be interpreted as experimentally confirmed evidence of poor absorption, cardiotoxicity, hepatotoxicity, or mutagenicity. Therefore, the present ADMET results should be considered preliminary and hypothesis-generating, and experimental studies are required to determine the actual pharmacokinetic and toxicological properties of compound **107**. Any subsequent structural optimization should be guided by the results of such experimental validation rather than by the computational predictions alone.

**Figure 5:**
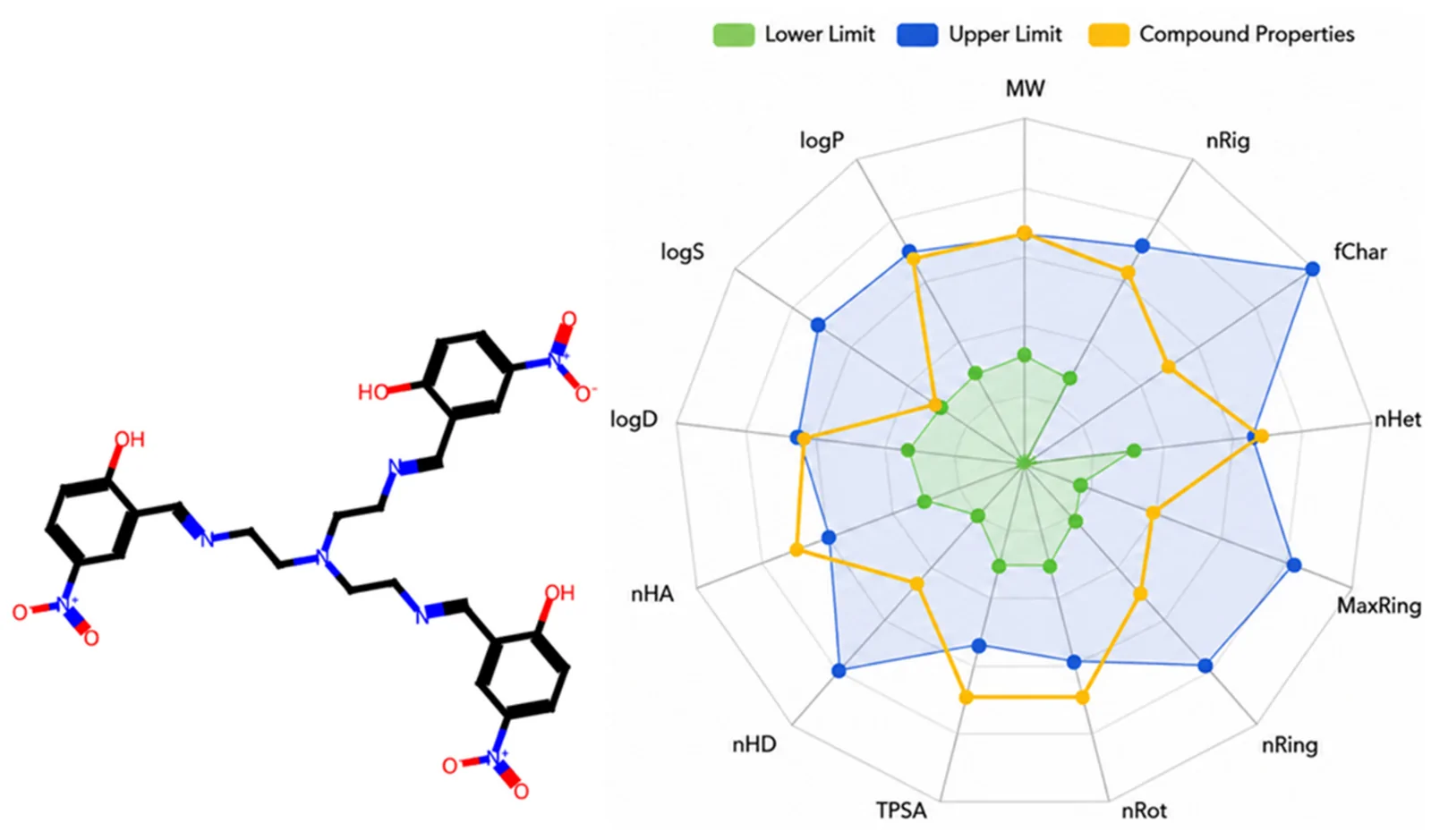
Structure, physicochemical and lipophilicity parameter images of compound **107**.

As shown in Figure 5, the radar plot provides a comparative visualization of the physicochemical and lipophilicity descriptors of compound **107** relative to their recommended lower and upper limits. The compound exhibits acceptable values for lipophilicity-related parameters, particularly logP and logD, indicating a balanced hydrophilic-lipophilic profile. However, deviations from the optimal ranges were observed for several molecular descriptors, including molecular weight (MW), topological polar surface area (TPSA), and the number of hydrogen bond acceptors (nHA). The elevated TPSA and nHA values suggest high polarity, which is generally associated with reduced membrane permeability and poor gastrointestinal absorption. Moreover, the structural representation of compound 107 reveals the presence of multiple heteroatoms and hydrogen-bonding functionalities, contributing to its increased polarity and relatively large molecular size. These structural features are consistent with the predicted HIA and Caco-2 permeability values and may contribute to the potentially limited oral absorption predicted by the computational model.

While Compound **107** demonstrated reduced metabolic viability and increased early apoptosis in HT-29 cells, these findings require careful interpretation. The high in silico AMES (0.999) and hepatotoxicity (0.81) values are theoretical predictions rather than absolute experimental proof. However, without a comparative assessment on a normal colonic epithelial line (e.g., CCD-841 CoN) to establish a Selectivity Index (SI), general toxicity cannot be experimentally ruled out. Therefore, until these computationally predicted risks are experimentally validated, the observed effects should be interpreted as preliminary metabolic viability-reducing and pro-apoptotic activity against HT-29 cells rather than as definitive evidence of selective anticancer activity.

### 2.8. Molecular Docking Interaction Analysis

In the present study, molecular docking analyses were performed to explore the potential interactions of the synthesized compound **107** against colon cancer-related molecular targets. In this context, vascular endothelial growth factor A (VEGFA, PDB ID: 3QTK) and vascular endothelial growth factor receptor 2 (VEGFR2, PDB ID: 2QU5), which play critical roles in tumor angiogenesis and progression in colon cancer, were selected as target proteins. The molecular interactions of compound **107** were investigated and compared with the reference chemotherapeutic agent Docetaxel. For in silico analyses, the clinically used chemotherapy drug DTX was specifically chosen as a comparative reference ligand. Although DTX is primarily known as a microtubule-stabilizing agent rather than a classical direct VEGFR2 inhibitor, it was specifically selected to maintain methodological consistency with the in vitro experimental design of this study. Because DTX serves as a standard positive control in cellular experiments (including immunofluorescence quantification of cell viability, apoptosis, and VEGFA/VEGFR2 suppression), its use as an in silico reference point provides a computational basis for comparing the predicted interaction profiles with the corresponding in vitro findings.

The binding affinity results obtained from docking simulations are summarized in Table 6. The findings revealed that compound **107** exhibited slightly lower predicted docking scores toward both target proteins compared to Docetaxel. Specifically, in the interaction with VEGFA (3QTK), compound **107** showed a binding energy of −8.60 kcal/mol, whereas Docetaxel exhibited a binding energy of −8.30 kcal/mol. Similarly, for VEGFR2 (2QU5), compound **107** demonstrated a binding score of −8.30 kcal/mol, which was slightly more favorable than that of Docetaxel (−8.00 kcal/mol). These results indicate that compound **107** may adopt potentially favorable predicted interaction modes within the investigated target binding regions. However, these findings should be considered preliminary and hypothesis-generating rather than evidence of experimentally confirmed target binding or inhibitory activity. For comparison, the interaction profile of Docetaxel (DTX) with VEGFA (PDB ID: 3QTK) was also examined in detail. DTX formed conventional hydrogen bonds with ARG-98, CYS-54, and ASN-55, with corresponding bond distances of 6.81, 3.77, and 4.96 Å, respectively. In addition, carbon–hydrogen bond interactions were detected with ASP-27 and SER-43, with distances of 5.66 and 4.41 Å, respectively. DTX also established a π–anion interaction with GLU-57 at a distance of 4.67 Å and a π–alkyl interaction with ILE-39 at 6.32 Å. Furthermore, van der Waals contacts with CYS-44, LEU-59, GLY-52, and LYS-100 contributed to the accommodation of DTX within the VEGFA binding region. Additional π–π T-shaped and π–π stacked interactions involving PHE-29 were observed at distances of 5.68 and 4.86 Å, respectively. Collectively, these interactions indicate that DTX was accommodated within the VEGFA binding region through a combination of hydrogen bonding, hydrophobic, π-related, and van der Waals contacts.

Similarly, the interaction profile of DTX with VEGFR2 (PDB ID: 2QU5) revealed several conventional hydrogen bonds involving ARG-1066, LEU-1049, ARG-1080, and TYR-1082, with bond distances of 5.47, 4.18/5.41, 4.48, and 6.76 Å, respectively. A π–cation interaction with ARG-1066 was also identified at a distance of 6.45 Å. In addition, DTX formed π–alkyl interactions with LEU-1069, PRO-1068, and ALA-844, with distances of 4.52, 5.40, and 6.06 Å, respectively. A π–anion interaction with ASP-1064 at 5.94 Å and an alkyl interaction with LEU-1049 at 4.75 Å were also detected. Van der Waals contacts involving ALA-881, ILE-1111, MET-1072, and ALA-1050 further contributed to the positioning of DTX within the VEGFR2 binding site. These results suggest that DTX may interact with the modeled VEGFR2 binding region through multiple non-covalent interaction types, although the specific interaction pattern differs from that observed for compound **107**.

The molecular docking interaction profiles of compound **107** and the reference drug Docetaxel with VEGFA (3QTK) and VEGFR2 (2QU5) were further investigated to elucidate the binding mechanisms at the amino acid level. The interaction parameters, including hydrogen bonds, hydrophobic contacts, π-interactions, and van der Waals forces, are summarized in Table 7 and Table 8. In the case of VEGFA (3QTK), compound **107** exhibited a more extensive interaction network compared to Docetaxel. The binding of compound **107** was stabilized by multiple conventional hydrogen bonds with key residues such as TYR-14, GLU-57, SER-43, ASN-55, CYS-54, and ARG-98, with bond lengths ranging from 3.67 to 5.92 Å. In addition, π–cation interactions with LYS-100 and ASP-56, as well as π–alkyl interactions involving PRO-99 and ILE-36/ILE-39, further contributed to the stability of the ligand–protein complex. Van der Waals interactions with residues including LYS-41, LEU-59, CYS-53, and GLU-60 also supported ligand accommodation within the active site. Notably, compound **107** additionally formed an unfavorable donor–donor interaction with LYS-101, indicating a localized steric or electrostatic constraint. In contrast, Docetaxel showed fewer interaction types and a relatively less dense binding profile. Although conventional hydrogen bonds were observed with ARG-98, CYS-54, and ASN-55, the overall interaction network was weaker, and no π–cation interactions were detected. The absence of such stabilizing interactions may contribute to the observed difference in the predicted docking scores; however, the relatively small score difference should not be interpreted as evidence of substantially stronger binding. For VEGFR2 (2QU5), compound **107** again demonstrated a richer interaction pattern. Strong hydrogen bonding interactions were observed with ASP-1129, ILE-1111, ARG-929, SER-925, SER-1104, and GLU-1102, with bond lengths between 3.54 and 5.16 Å. Additional stabilizing interactions included π–cation bonding with LYS-1070, π–sigma interactions with ALA-1103, and π–alkyl contacts with LEU-1067. Furthermore, a π–donor hydrogen bond with SER-1104 contributed to the stabilization of the ligand within the active site. These multiple interaction types suggest a potentially compatible binding orientation of compound **107** within VEGFR2. By comparison, Docetaxel exhibited a less complex interaction profile with VEGFR2, forming fewer hydrogen bonds with residues such as ARG-1066, LEU-1049, ARG-1080, and TYR-1082. Although some hydrophobic and π-related interactions (e.g., π–alkyl and π–anion interactions) were present, the overall interaction density was lower than that of compound **107**. Collectively, these results suggest that compound **107** forms a somewhat more diverse predicted interaction pattern with both VEGFA and VEGFR2 compared to Docetaxel. This enhanced binding profile may contribute to the slightly lower docking scores obtained for compound **107**; however, the findings should be regarded as preliminary and hypothesis-generating and require further experimental validation.

**Figure 6:**
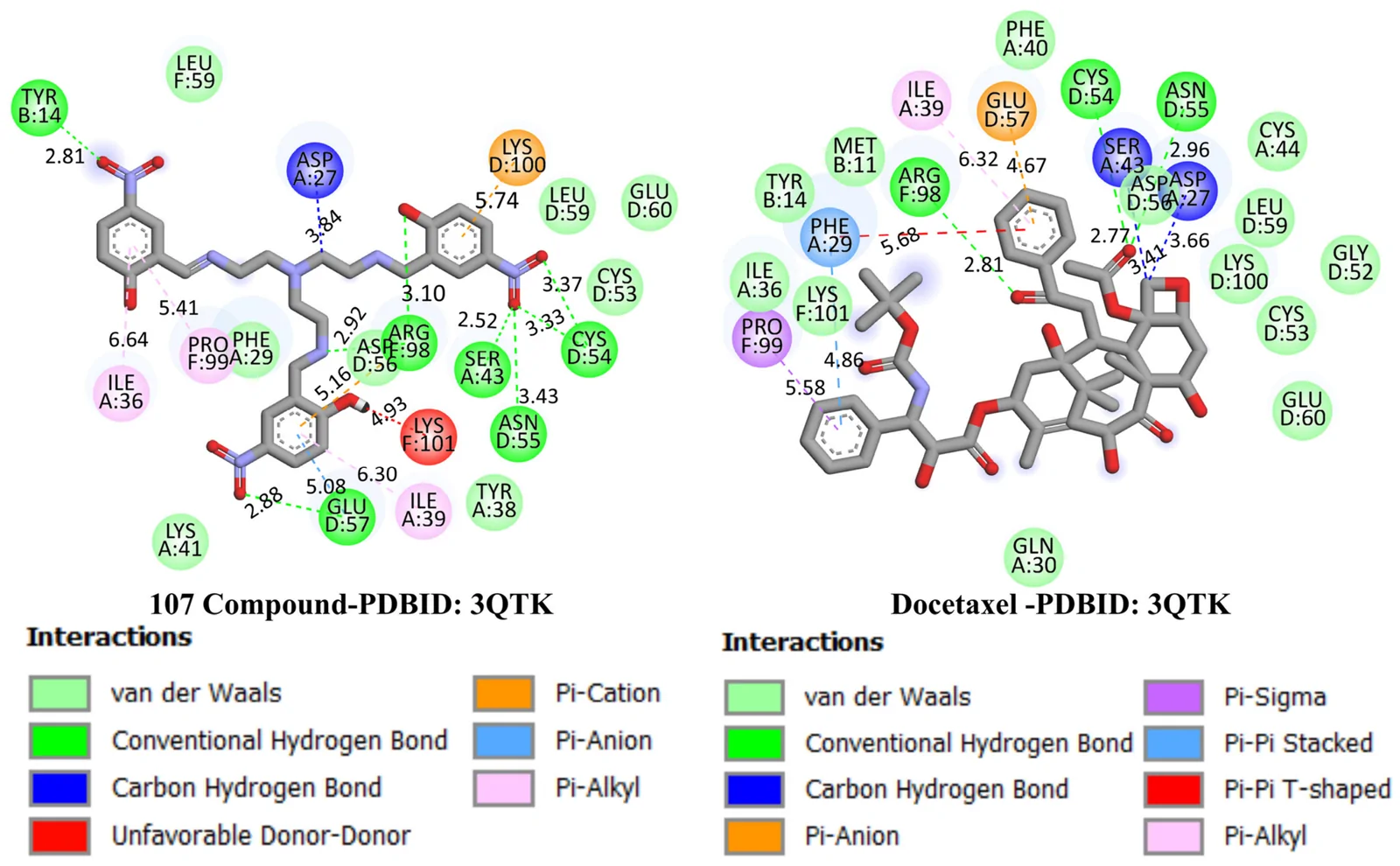
Two-Dimensional Mode view of **107**, DTX Compounds and PDB ID: 3QTK enzyme.

**Figure 7:**
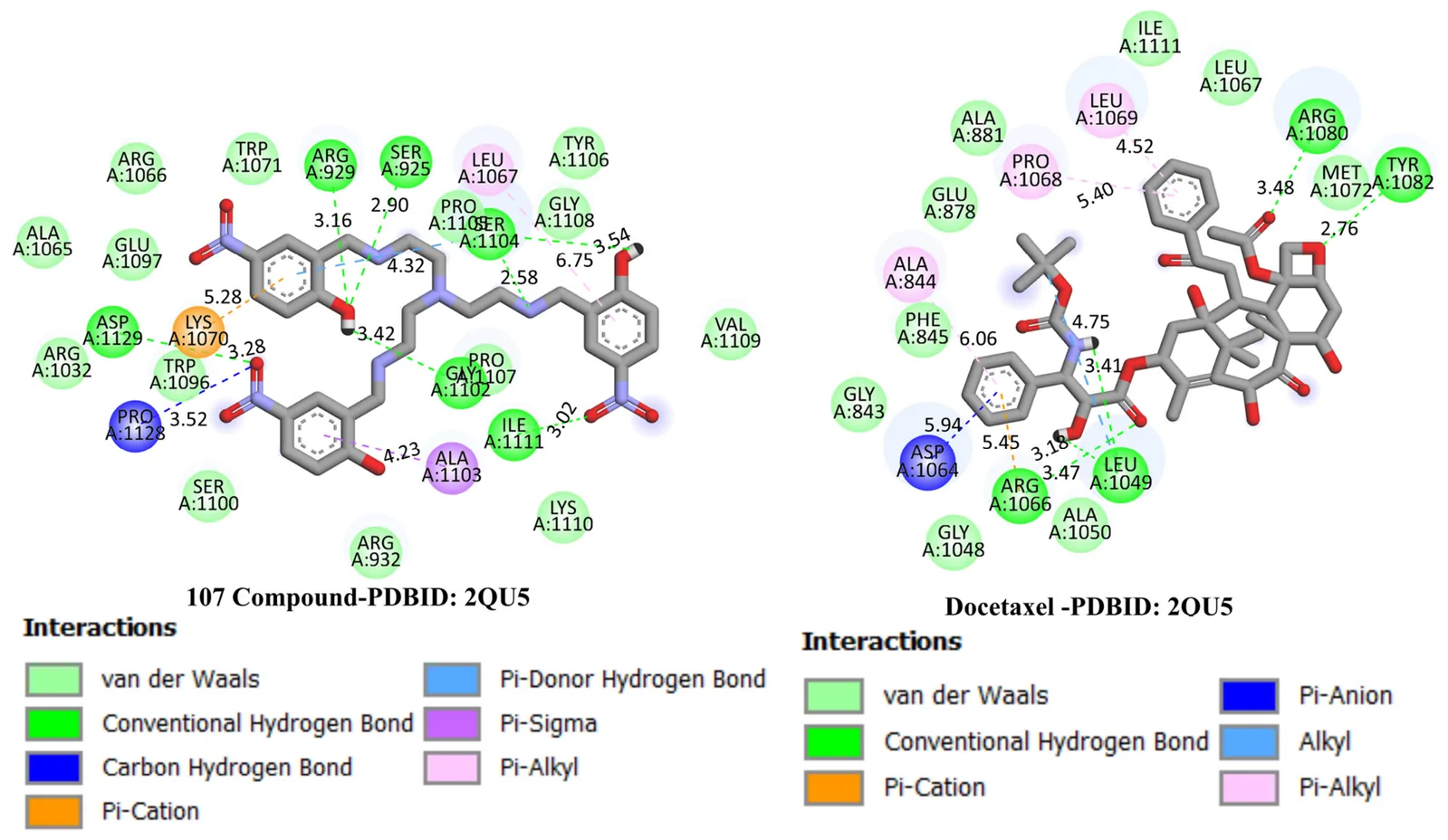
Two-Dimensional Mode view of **107**, DTX Compounds and PDB ID: 2QU5 enzyme.

The 2D interaction profiles of compound **107** and Docetaxel with VEGFA (3QTK) and VEGFR2 (2QU5) are illustrated in Figure 6 and Figure 7, respectively. These figures provide a comprehensive visualization of ligand–protein binding modes, highlighting the types and spatial distribution of molecular interactions within the active sites.

As shown in Figure 6, compound **107** establishes a dense and well-organized interaction network within the VEGFA (3QTK) binding pocket. The ligand is stabilized through multiple conventional hydrogen bonds with key residues such as TYR-14, GLU-57, SER-43, ASN-55, CYS-54, and ARG-98, consistent with the interaction data summarized in Table 7. In addition, Figure 6 clearly demonstrates the presence of π–cation interactions with LYS-100 and ASP-56, as well as π–alkyl contacts involving PRO-99 and ILE-36/ILE-39, which contribute significantly to the stabilization of the ligand within the active site. Van der Waals interactions distributed across residues such as LYS-41, LEU-59, CYS-53, and GLU-60 further support the binding affinity of compound **107**. In contrast, the Docetaxel-3QTK complex in Figure 6 shows a comparatively less dense interaction pattern, characterized by fewer hydrogen bonds and a reduced number of π-type interactions, indicating a different and comparatively less diverse predicted interaction pattern. Similarly, Figure 7 illustrates the binding modes of compound **107** and Docetaxel within the VEGFR2 (2QU5) active site. Compound **107** exhibits a strong and multi-point interaction profile, forming stable hydrogen bonds with residues ASP-1129, ILE-1111, ARG-929, SER-925, SER-1104, and GLU-1102. The figure further highlights additional stabilizing interactions, including π–cation bonding with LYS-1070, π–sigma interaction with ALA-1103, and π–alkyl interactions with LEU-1067. These interactions suggest that compound **107** may adopt a potentially favorable orientation within the VEGFR2 binding pocket, consistent with the interaction parameters reported in Table 8. Conversely, the Docetaxel–VEGFR2 complex in Figure 7 displays fewer hydrogen bonding interactions and a relatively limited hydrophobic interaction network, suggesting a different predicted binding conformation compared to compound **107**.

Overall, Figure 6 and Figure 7 collectively indicate that compound **107** forms a different and somewhat more diverse predicted interaction pattern with both VEGFA (3QTK) and VEGFR2 (2QU5) compared to Docetaxel. The higher diversity of predicted hydrogen-bond and π-related interactions is consistent with the slightly lower docking scores obtained for compound **107**. However, the relatively small difference of approximately 0.30 kcal/mol should not be interpreted as evidence of superior binding affinity or biological activity. These findings suggest that compound **107** may warrant further investigation in relation to angiogenesis-associated molecular targets in colon cancer.

As presented in Table 6, the calculated Root Mean Square Deviation (RMSD) values for both Compound **107** and the reference drug DTX against VEGFA (3QTK) and VEGFR2 (2QU5) range between 1.50 Å and 1.80 Å. Since the obtained RMSD values are below the commonly used 2.0 Å criterion, they provide supportive information regarding the similarity of the predicted poses to the corresponding reference conformations. These low RMSD values should not be interpreted as evidence of binding stability, superior binding affinity, or biological activity. Rather, they provide supportive information regarding the pose similarity under the applied docking conditions, thereby providing supportive information for our in silico methodology.

### 2.9. Cell Culture Study Results

#### 2.9.1. MTT-Based Metabolic Viability Results

**Figure 8:**
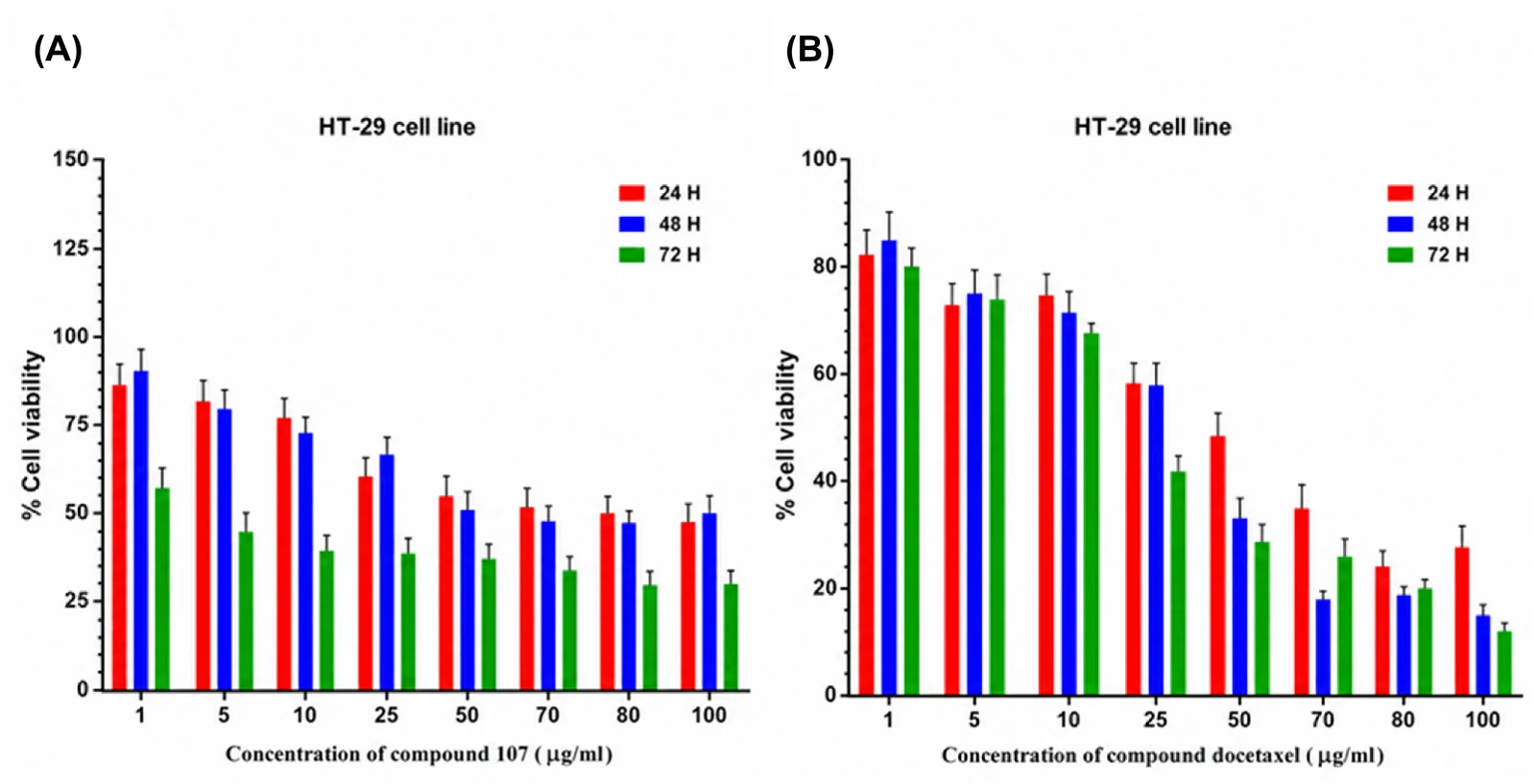
Effects of Compound **107** and DTX on HT-29 MTT-based metabolic viability. Metabolic viability was evaluated using the MTT assay following treatment with increasing concentrations (1, 5, 10, 25, 50, 70, 80, and 100 µg/mL) of (**A**) Compound **107** and (**B**) DTX for 24, 48, and 72 h. Data are presented as mean ± SD.

The effects of Compound **107** and DTX on the MTT-based metabolic viability of HT-29 cells were evaluated after 24-, 48-, and 72-h incubation periods. In both applications, a decrease in MTT-based metabolic viability was observed with increasing concentrations and incubation times (Figure 8). The decrease in metabolic viability was more pronounced with Compound **107**, especially after 72 h of incubation. With DTX application, a significant and gradual decrease in cell viability was observed as the concentration of DTX increased. These findings were also consistent with the IC_50_ values (Table 9). For Compound **107**, the IC_50_ values were 32.91, 19.96, and 17.51 µg/mL at 24, 48, and 72 h, respectively, whereas for DTX, these values were >80.79, 61.26, and 37.86 µg/mL, respectively. The decrease in IC_50_ values with increasing incubation time for both compounds indicated a greater reduction in MTT-based metabolic viability over time. Under the specific in vitro experimental conditions used in this study, Compound **107** showed lower IC_50_ values than DTX at all evaluated incubation times, indicating that a lower concentration of Compound **107** was required to reduce HT-29 MTT-based metabolic viability by 50%. This observation should not be interpreted as evidence that Compound **107** is generally more effective or therapeutically superior to DTX.

#### 2.9.2. Expression in HT29 Cell Groups

In the HT29 cell experiments, both DTX and Compound **107** treatment reduced the relative expression of *VEGFA* and *KDR* compared with the control group. Relative VEGFA expression was 0.53-fold in the DTX group and 0.75-fold in the Compound **107** group, whereas relative *KDR* expression was 0.24-fold and 0.74-fold, respectively. Comparisons performed on ΔCt values using two-way ANOVA followed by Dunnett’s multiple-comparisons test showed that all treatment groups differed significantly from the control group, with all adjusted *p* < 0.0001 (Figure 1B).

#### 2.9.3. Cell Cycle Analysis Results

**Figure 9:**
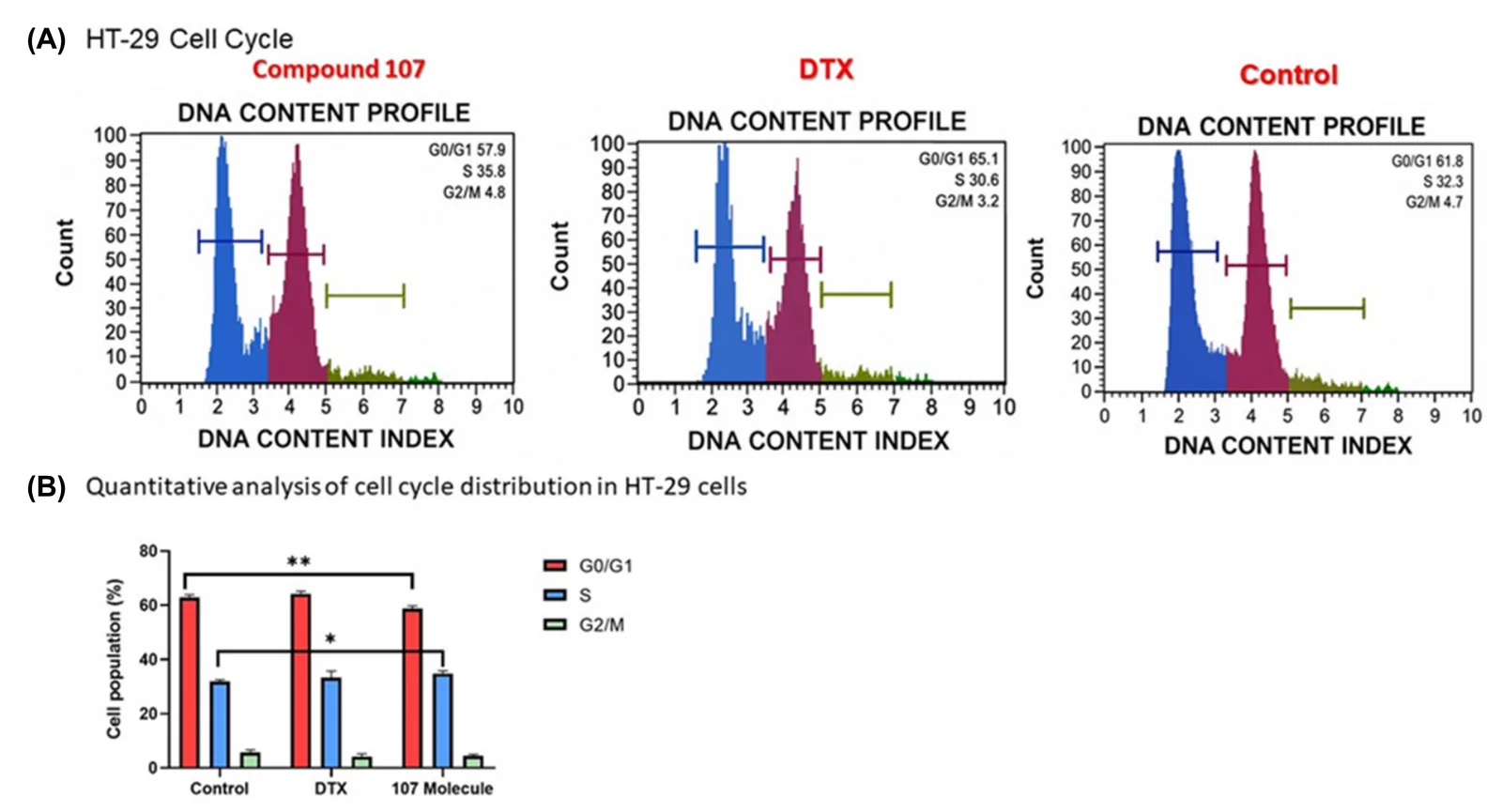
Effects of Compound **107** and DTX on cell cycle distribution in HT-29 cells. (**A**) Representative DNA content profiles. (**B**) Quantitative distribution of cells in G0/G1, S, and G2/M phases. * *p* < 0.05, ** *p* < 0.01 versus control.

The G0/G1 population was significantly lower in the Compound **107** group than in the control group (** *p* = 0.0012), whereas the S-phase population was significantly higher (* *p* = 0.0176). No significant differences were observed between the control and DTX groups or in the G2/M population (Figure 9A,B).

#### 2.9.4. Annexin V Binding Assay Results

**Figure 10:**
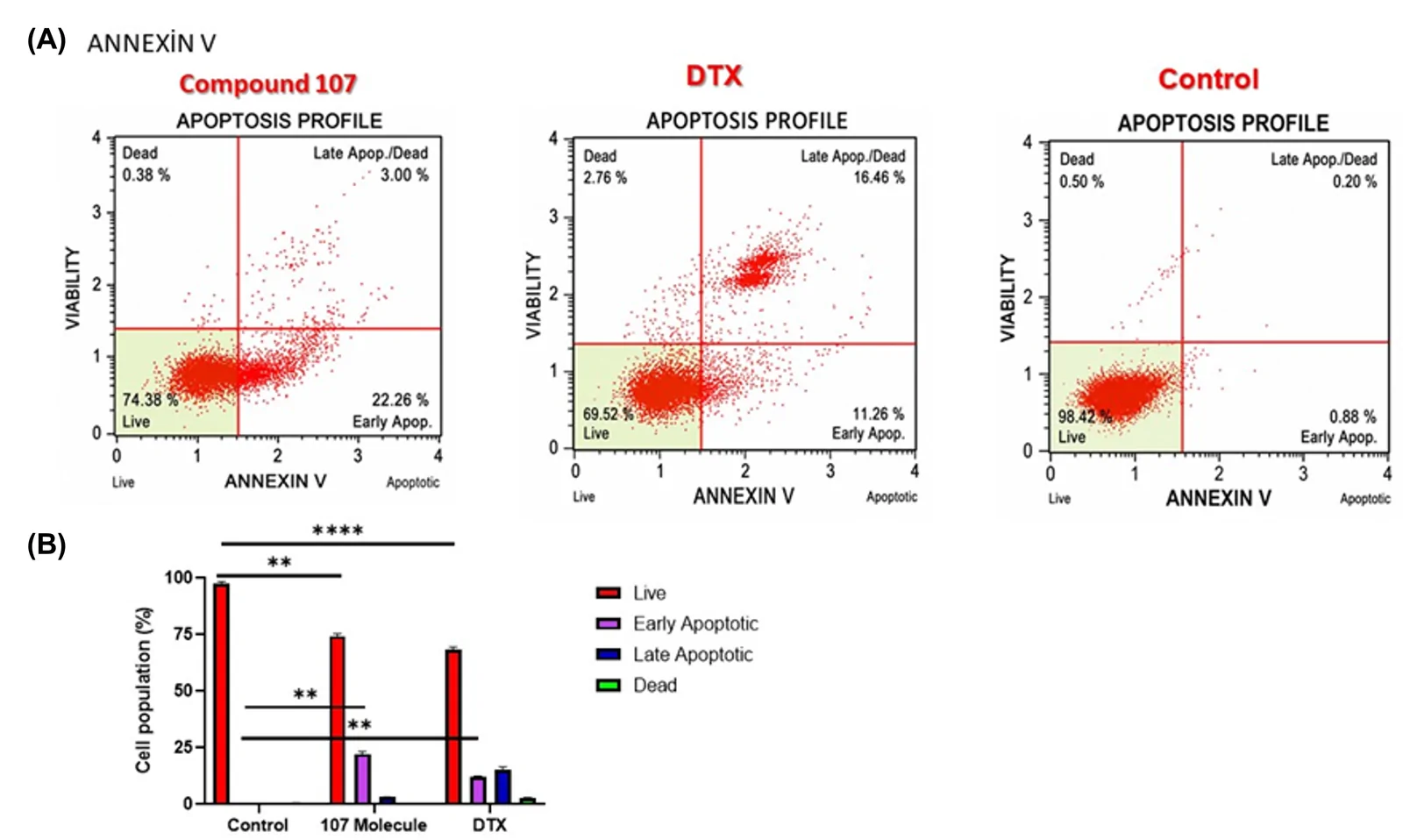
Effects of Compound **107** and DTX on apoptosis in HT-29 cells. (**A**) Representative Annexin V/Dead Cell profiles. (**B**) Quantitative analysis of viable, early apoptotic, late apoptotic, and dead cell populations. Statistical comparisons were performed using two-way ANOVA followed by Dunnett’s multiple-comparisons test, with the control group used as the reference. ** *p* < 0.0001, **** *p* < 0.0001. versus control; ns, not significant.

The proportion of live cells was significantly decreased in both the Compound **107** and DTX groups compared with the control group (both adjusted *p* < 0.0001). Early apoptotic cell proportions were significantly increased following both Compound **107** and DTX treatment (both adjusted *p* < 0.0001). Similarly, late apoptotic cell proportions were significantly increased in both treatment groups compared with the control group (both adjusted *p* < 0.0001). For the dead-cell population, no significant difference was observed between the Compound **107** and control groups (adjusted *p* = 0.9999), whereas the DTX group showed a significant increase compared with the control group (adjusted *p* < 0.0001) (Figure 10A,B).

#### 2.9.5. Immunofluorescence Analysis of VEGFA and VEGFR2

**Figure 11:**
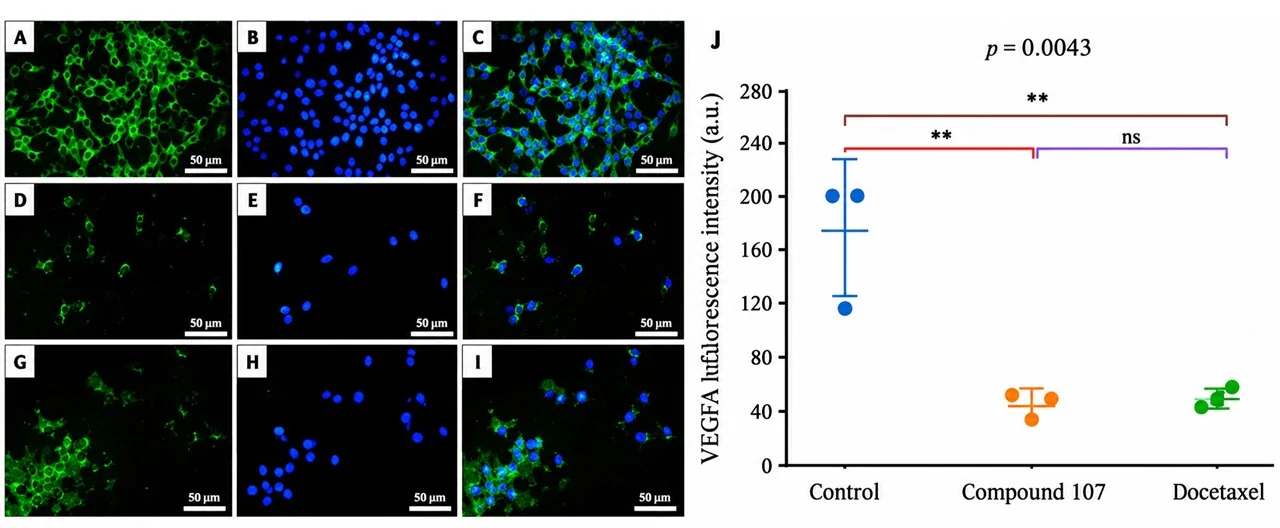
Immunofluorescence analysis of VEGFA expression in HT-29 colorectal cancer cells. Representative images showing VEGFA immunofluorescence (green), DAPI nuclear counterstaining (blue), and merged fluorescence in untreated Control (**A**–**C**), Compound **107**-treated (**D**–**F**), and DTX-treated (**G**–**I**) HT-29 cells. VEGFA immunoreactivity was predominantly localized to the cytoplasmic compartment and was markedly reduced following treatment with either Compound **107** or DTX. All images were acquired using a ×40 objective under identical microscope settings. Scale bars = 20 μm. (**J**) Quantitative analysis of cellular VEGFA fluorescence using the corrected total cell fluorescence (CTCF) method. Cellular regions of interest were generated by threshold-based semi-automated segmentation of the Alexa Fluor 488 channel, with DAPI staining used to identify cell nuclei. CTCF was calculated as Integrated Density − (Cell Area × Mean Background Fluorescence). Each point represents the mean value obtained from one independent experiment after averaging three randomly selected, non-overlapping microscopic fields from the corresponding well (n = 3 independent experiments per group). Data are presented as mean ± SD. Statistical significance was evaluated using one-way ANOVA followed by Tukey’s multiple-comparisons test. A significant overall difference was detected among the groups (*p* = 0.0043). VEGFA fluorescence was significantly lower in the Compound **107**- and DTX-treated groups than in the untreated Control group (both *p* < 0.01), whereas no significant difference was observed between the Compound **107** and DTX groups (*p* > 0.05). ** indicates *p* < 0.01; ns, not significant.

**Figure 12:**
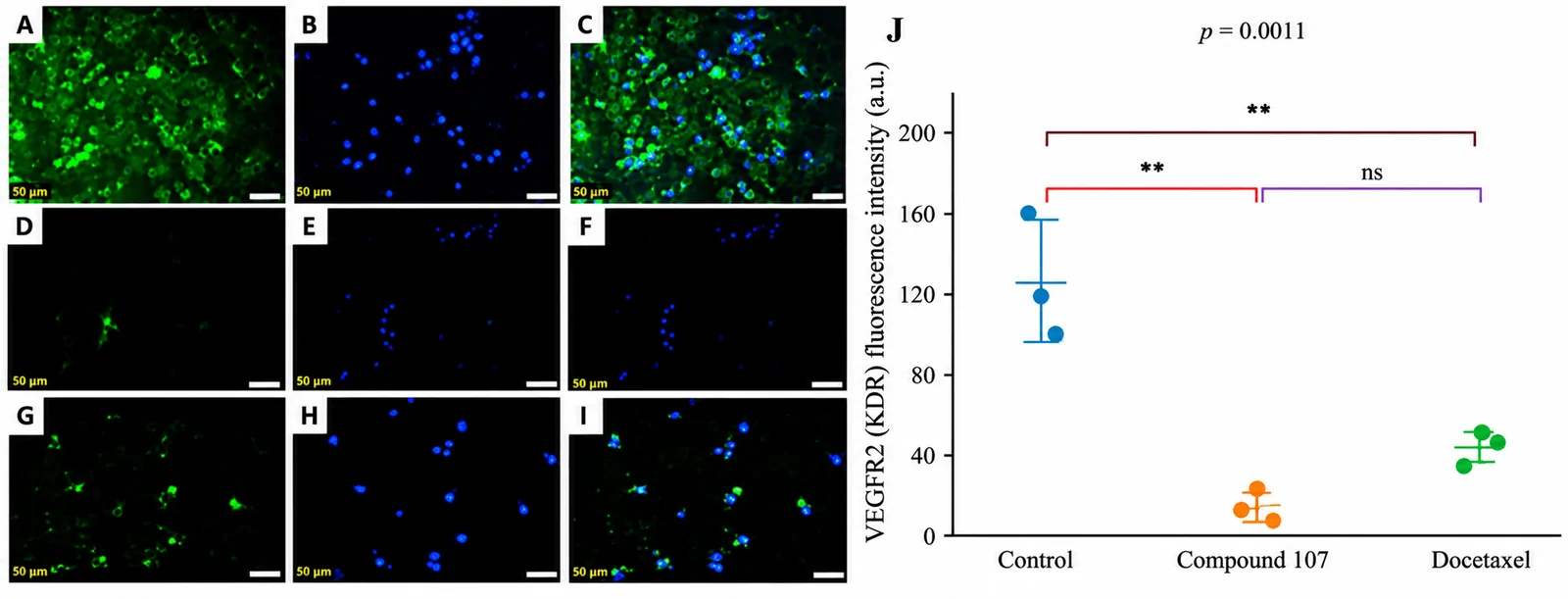
Immunofluorescence analysis of VEGFR2/KDR expression in HT-29 colorectal cancer cells. Representative images showing VEGFR2 immunofluorescence (green), DAPI nuclear counterstaining (blue), and merged fluorescence in untreated Control (**A**–**C**), Compound **107**-treated (**D**–**F**), and DTX-treated (**G**–**I**) HT-29 cells. Anti-VEGFR2 antibody-associated fluorescence showed an apparent membrane-associated and cytoplasmic distribution and was reduced following treatment with either Compound **107** or DTX. All images were acquired using a ×40 objective under identical microscope settings. Scale bars = 20 μm. (**J**) Quantitative analysis of cellular VEGFR2 fluorescence using the corrected total cell fluorescence (CTCF) method. Cellular regions of interest were generated by threshold-based semi-automated segmentation of the Alexa Fluor 488 channel, with DAPI staining used to identify cell nuclei. CTCF was calculated as Integrated Density − (Cell Area × Mean Background Fluorescence). Each point represents the mean value obtained from one independent experiment after averaging three randomly selected, non-overlapping microscopic fields from the corresponding well (n = 3 independent experiments per group). Data are presented as mean ± SD. Statistical significance was evaluated using one-way ANOVA followed by Tukey’s multiple-comparisons test. A significant overall difference was detected among the groups (*p* = 0.0011). VEGFR2 fluorescence was significantly lower in the Compound **107**- and DTX-treated groups than in the untreated Control group (both *p* < 0.01), whereas no significant difference was observed between the Compound **107** and DTX groups (*p* > 0.05). ** indicates *p* < 0.01; ns, not significant.

Representative immunofluorescence images demonstrated strong and widespread cytoplasmic VEGFA immunoreactivity in untreated HT-29 cells. In contrast, VEGFA fluorescence intensity was markedly reduced following treatment with either Compound **107** or DTX (Figure 11A–I). Quantitative CTCF analysis revealed a significant overall difference among the Control, Compound **107**, and DTX groups (one-way ANOVA, *p* = 0.0043; Figure 11J). Tukey’s multiple-comparisons test demonstrated that VEGFA fluorescence intensity was significantly lower in both the Compound **107**- and DTX-treated groups than in the untreated Control group (both *p* < 0.01). No statistically significant difference in VEGFA immunofluorescence intensity was detected between the Compound **107** and DTX groups (*p* > 0.05). In untreated HT-29 cells, staining with the anti-VEGFR2 antibody showed an apparent membrane-associated and cytoplasmic fluorescence pattern. Treatment with either Compound **107** or DTX resulted in a marked reduction in VEGFR2 fluorescence intensity (Figure 12A–I). Quantitative CTCF analysis demonstrated a significant overall difference among the experimental groups (one-way ANOVA, *p* = 0.0011; Figure 12J). Tukey’s multiple-comparisons test showed that VEGFR2 fluorescence intensity was significantly lower in both the Compound **107**- and DTX-treated groups than in the untreated Control group (both *p* < 0.01). No statistically significant difference was observed between the Compound **107** and DTX groups (*p* > 0.05).

## 3. Discussion

This study evaluated the status of the VEGFA and VEGFR2 signaling pathway in CRC using clinical tissue samples and assessed the potential effects of Compound **107**, an azomethine/Schiff base compound, on this pathway using a combination of clinical, in vitro, and in silico approaches. The main findings of the study were as follows: increased *VEGFA* and *KDR* gene expression in colorectal tumor tissues compared to paired non-neoplastic tissues; confirmation of this increase at the immunohistochemical protein level; and reduction in cell viability, increased early apoptosis, altered cell cycle distribution, and suppression of *VEGFA* and *KDR* expression in HT-29 cells by Compound 107 application. Furthermore, molecular docking analysis showed that compound **107** exhibited favorable binding profiles to VEGFA and VEGFR2, providing a complementary molecular framework to the experimental findings. However, the docking results should not be considered direct experimental evidence of target binding or VEGFR2 inhibition.

Angiogenesis is a crucial biological process in the growth, invasion, and metastatic progression of CRC, and the VEGF/VEGFR system is a key regulator of angiogenesis. Yang et al. highlighted that VEGF-mediated signaling is central to CRC angiogenesis and that primary or acquired resistance mechanisms remain a significant challenge despite the clinical importance of anti-angiogenic therapies [20]. Similarly, Jacobsen et al. reported that the CRC vascular system exhibits significant phenotypic and functional heterogeneity, and that pathological vascularity is closely related to impaired perfusion, altered barrier function, and the immunological microenvironment [9]. These findings suggest that the VEGFA–VEGFR2/CDR system should be evaluated not only in terms of classical angiogenesis but also in terms of the broader biological characteristics of the tumor microenvironment.

In our study, the 1.98-fold increase in VEGFA expression in tumor tissues compared to matched non-neoplastic tissues is consistent with current molecular data demonstrating the importance of the VEGF family in CRC biology. Yang et al. performed multi-omics analysis and revealed the expression characteristics, prognostic relationships, and connections with the tumor microenvironment of the VEGF family in colon adenocarcinoma [1]. In addition, Long et al. showed that extracellular vesicles released from CRC cells can increase endothelial cell proliferation, migration, and tube formation via JAK/STAT3/VEGFA signaling [21]. Ma et al. reported that the LINC01106/miR-449b-5p/VEGFA regulatory system plays a role in CRC vascularization and that this process can be regulated by YTHDF1-related epitranscriptomic mechanisms [5]. These studies show that VEGFA expression is at the intersection of numerous molecular mechanisms in CRC.

The mechanisms related to PI3K/AKT are also important in the regulation of VEGFA levels. Xu et al. showed that USP13 reduces VEGFA expression and suppresses CRC angiogenesis by regulating the PTEN-AKT pathway [6]. In particular, the reduction in VEGFA levels and suppression of endothelial migration, invasion, and tube formation by USP13 overexpression reveal the link between VEGFA and intracellular proliferative signals and the vascular microenvironment of the tumor. Therefore, the increase in VEGFA observed in tumor tissues in our study should be considered as part of a broader angiogenic signaling network reorganized in CRC rather than a single molecular change.

Another important finding of our study is that *KDR* gene expression was approximately 2.02-fold higher in tumor tissues than in paired non-neoplastic tissues. In recent years, single-cell transcriptomic approaches have revealed the detailed role of *KDR* in the CRC microenvironment. Xie et al. examined endothelial cell heterogeneity in CRC and showed that KDR is particularly enriched in angiogenic cells and that the VEGFA–KDR–ESM1 positive feedback loop supports tumor cell proliferation and migration along with angiogenesis [4]. This finding is consistent with the co-increase in VEGFA and *KDR* expression in tumor tissue in our study.

The function of VEGFR2 in CRC does not appear to be limited to the vascular endothelium. Tsumuraya et al. showed that VEGFR2 expression is found in a significant proportion of effector regulatory T cells in the tumor microenvironment of advanced CRC patients [3]. In the same study, the positive correlation between *KDR* expression and Treg-related gene signatures and the ability of VEGFR2 inhibition to affect immunosuppressive properties suggest that the VEGF/VEGFR2 pathway may also be functional in the tumor immune microenvironment. Therefore, associating the increase in VEGFR2/KDR expression solely with tumor vessel formation may be an incomplete approach.

In our study, it is also important that the RT-qPCR results for VEGFA and VEGFR2/KDR are supported by immunohistochemical findings. The immunoreactivity of both proteins was significantly higher in colorectal adenocarcinoma tissues than in matched non-neoplastic tissues. The concordance between transcript measurements and immunohistochemical findings supports increased VEGFA and VEGFR2 expression-associated signals in CRC tissues, but does not establish activation of the VEGFA/VEGFR2 signaling pathway. However, since the current study was not designed for prognostic purposes and long-term survival data were not evaluated, it is not appropriate to conclude that VEGFA or VEGFR2/*KDR* are prognostic biomarkers.

The clinical significance of the VEGF/VEGFR system is also evident in the role of anti-angiogenic therapies in metastatic colorectal cancer (CRC). In the SUNLIGHT phase III study, Prager et al. demonstrated that the combination of trifluridine/tipiracil and bevacizumab extended overall and progression-free survival in refractory metastatic CRC compared with trifluridine/tipiracil treatment alone [22]. These clinical results support the importance of therapeutically targeting angiogenesis in advanced CRC patients. However, the development of resistance to anti-VEGF therapy is a significant limitation. Liu et al. showed that gamma-synuclein can contribute to bevacizumab resistance by maintaining VEGFR2 activation and angiogenesis [23]. These results suggest that neutralization of the VEGF ligand may not completely inhibit VEGFR2 signaling in all cases and that alternative regulatory mechanisms associated with VEGFR2 may contribute to treatment resistance.

In this context, the results obtained with compound **107** are noteworthy. In HT-29 cells, both Compound **107** and DTX reduced cell viability in a concentration- and time-dependent manner, and IC_50_ values were determined. The decrease in IC_50_ values with increasing incubation time in both applications indicates a greater reduction in MTT-based metabolic viability over time. The fact that compound **107** showed lower IC_50_ values at all time points indicates that, under the experimental conditions in our study, it required lower concentrations than DTX to reduce HT-29 MTT-based metabolic viability by 50%. However, this result should not be interpreted as indicating that compound **107** is generally stronger or clinically more effective than DTX.

The azomethine/Schiff base structure of Compound **107** is also important in terms of the cytotoxic findings obtained. Compounds containing Schiff bases are increasingly being investigated in anticancer drug research because they can be easily chemically modified and interact with different cellular targets [24,25]. However, the biological activity of these compounds can vary significantly depending on their molecular structure, substituent properties, lipophilicity, and interactions with target proteins. Therefore, it is not appropriate to directly generalize the results obtained with compound **107** to other compounds in the same chemical class.

There is also experimental evidence regarding the anticancer activities of some Schiff base derivatives related to VEGFR2 activity. Abd El-Lateef et al. reported that some derivatives of Schiff base-benzimidazole hybrids showed significant VEGFR2 tyrosine kinase inhibitory activity, altered the G1 and S phases of the cell cycle, and had a pro-apoptotic effect as demonstrated by Annexin V [11]. Another study on isatin-based Schiff base derivatives reported that some compounds exhibited antiproliferative and anti-angiogenic properties and displayed activity associated with VEGFR2 inhibition [26]. Furthermore, sulfonamide-bound Schiff base derivatives have been shown to suppress VEGFR2 kinase activity, and some active compounds have demonstrated significant pro-apoptotic effects [27]. While these studies provide a scientific basis for the possible relationship between the structural class of Compound **107** and VEGFR2, they do not prove that Compound **107** is a direct VEGFR2 inhibitor. In our study, the application of Compound **107** significantly increased the rate of early apoptosis in HT-29 cells, suggesting that the decrease in cell viability may be at least partly related to programmed cell death. Studies on novel VEGFR2-targeting small molecules have shown that cytotoxic effects can occur in conjunction with Annexin V positivity and cell cycle changes [28]. Similarly, Taştemur et al. showed that a different experimental compound could increase the apoptotic response while suppressing growth-related signaling pathways in HT-29 cells [29]. In this respect, the Annexin V results in our study constitute an independent cellular finding supporting the decrease in cell viability determined by the MTT assay.

The application of compound **107** also altered the cell cycle distribution; the ratio of cells in the G0/G1 phase decreased, whereas the ratio of cells in the S phase increased significantly. This suggests that Compound **107** may affect cellular processes associated with DNA synthesis and S-phase progression. The fact that VEGFR2-targeted Schiff base-benzimidazole derivatives have been shown to induce cell cycle changes in the G1 and S phases [11] is consistent with our results. However, since cyclins, CDKs, or cell cycle checkpoint proteins were not evaluated in our study, a definitive interpretation of the specific molecular mechanism underlying the increase in the S-phase cannot be made. In contrast, DTX treatment did not induce a statistically significant change in the G2/M population under the experimental conditions used in this study. Although G2/M arrest is a well-established cellular effect of docetaxel [30], this expected response was not observed in our experiments. Therefore, the DTX-related cell-cycle findings should be interpreted with caution, and DTX should not be considered a validated positive control for G2/M arrest in the present experimental setting. The absence of the expected DTX-induced G2/M accumulation represents a limitation of the present study.

One of the most important findings of our study in terms of signaling mechanisms was the significant decrease in VEGFA and *KDR* gene expression after Compound **107** application compared to the control group. Immunofluorescence analyses also showed significantly reduced VEGFA fluorescence and anti-VEGFR2 antibody-associated fluorescence following Compound **107** treatment. However, the reduction in KDR/VEGFR2 expression does not establish VEGFR2 as a direct molecular target of Compound **107**. This reduction may also be secondary to cellular stress, reduced proliferation, apoptosis, or other upstream regulatory effects induced by the treatment. Furthermore, no statistically significant difference in VEGFA or VEGFR2 immunofluorescence intensity was detected between the Compound **107** and DTX groups under these experimental conditions. However, this finding does not imply that the mechanisms or overall anticancer activities of the two compounds are equivalent. A further limitation concerns the specificity of the anti-VEGFR2 immunofluorescence signal in HT-29 cells. The observed fluorescence and its reduction following treatment should be interpreted cautiously because receptor identity and localization were not independently confirmed by *KDR* knockdown, peptide-blocking controls, or Western blotting. Accordingly, these findings represent changes in anti-VEGFR2 antibody-associated fluorescence rather than definitive evidence of changes in membrane-localized VEGFR2 abundance or receptor activity. Validation incorporating HUVEC positive controls and complementary specificity assays is required.

The immunohistochemical analysis was also limited by the assessment of one selected microscopic field per tissue specimen. Although field selection followed predefined morphological and technical criteria and was performed blinded to tissue identity and clinicopathological information, a single field may not adequately capture intratumoral heterogeneity. The resulting IOD measurements should therefore be interpreted as semi-quantitative estimates of staining in the selected fields rather than comprehensive measurements of whole-section immunoreactivity. Multiple-field sampling or whole-section digital analysis, together with compartment-specific assessment, would provide a more representative evaluation.

Our molecular docking results provide a structural perspective that complements our observed cellular findings. Compound **107** exhibited highly favorable binding energies to VEGFA (−8.60 kcal/mol) and VEGFR2 (−8.30 kcal/mol), exhibiting comparable energetic profiles to the reference drug DTX. In structure-based drug design targeting VEGFR2, strong electrostatic interactions and hydrogen bonding networks are classically known to be critical for stabilizing ligand–receptor complexes [31,32]. It is important to note that, in the VEGFR2 (2QU5) model, Compound **107** and DTX interact with residues located at the C-terminal region of the catalytic domain (such as ASP-1129, LYS-1070, and LEU-1067), which are situated outside the canonical ATP-binding pocket (defined by the hinge region Cys919 and the DFG motif Asp1046). Consequently, Compound **107** does not appear to act as a classical ATP-competitive kinase inhibitor. Instead, it successfully establishes a dense and highly stable electrostatic interaction network at a non-canonical, potentially allosteric surface pocket.

While this stable surface binding cannot support claims of direct kinase catalytic inhibition, it establishes a theoretical framework that corresponds with our experimental in vitro data. The predicted interactions of Compound **107** with VEGFR2 and VEGFA provide a possible structural hypothesis that may be related to the observed reductions in gene expression and protein fluorescence intensity; however, these findings do not establish direct target engagement. Therefore, the cytotoxic and pro-apoptotic effects of Compound **107** were accompanied by reduced VEGFA and VEGFR2 expression; however, these findings do not demonstrate direct inhibition of the VEGFA/VEGFR2 signaling pathway. However, future experimental evaluations, including direct ligand–protein binding assays and allosteric functional studies, remain necessary to precisely define this mechanism [31,32].

The fact that Compound **107** forms numerous hydrogen bonds with VEGFR2, in addition to π–cation, π–sigma, and π–alkyl interactions, theoretically indicates favorable ligand–protein localization. However, the docking score is not equivalent to biological inhibitory power. Studies evaluating some Schiff derivatives have shown that docking results need to be supported by functional analyses, such as VEGFR2 kinase inhibition experiments [11,26,27]. Therefore, future experimental evaluations of VEGFR2 kinase activity, phospho-VEGFR2 levels, and direct ligand–protein binding for Compound **107** are necessary.

The ADMET results for compound **107** necessitate a more cautious assessment of the translational potential of the molecule. Its high molecular weight and topological polar surface area, along with its estimated low intestinal absorption and CaCO2 permeability, indicate limitations, particularly in terms of oral bioavailability. More importantly, the estimated risks of hERG blockade, hepatotoxicity, and AMES mutagenicity limit its direct identification as a therapeutic candidate with its current structure. Recent studies and reviews evaluating the anticancer potential of some Schiff compounds also emphasize that biological activity alone is insufficient and that pharmacokinetic properties and toxicity profiles should be considered together in the selection of candidate molecules [24,29,33]. Therefore, it would be more appropriate to evaluate Compound **107** at this stage not as a clinical drug candidate but as an experimental compound exhibiting antitumor activity and open to structural optimization.

## 4. Materials and Methods

### 4.1. Patient and Control Populations

A total of 32 patients diagnosed with CRC who were admitted to the Department of General Surgery at Sivas Cumhuriyet University Faculty of Medicine Research and Application Hospital were included in the study. The diagnosis of CRC was confirmed by histopathological examination of tissue specimens obtained during surgical resection. Based on the pathological evaluation, surgically resected tumor tissues were designated as the tumor group, whereas tissues obtained from the surgical margins adjacent to the tumor and histologically confirmed to be non-neoplastic were designated as the tumor-adjacent non-neoplastic tissue group. Thus, paired tumor and tumor-adjacent non-neoplastic tissue samples were obtained from each patient. No restrictions regarding age or sex were applied during patient recruitment. The clinical cohort consisted of 32 patients recruited from a single center. Importantly, the comparator samples were matched tumor-adjacent tissues histologically confirmed to be non-neoplastic from the same patients, rather than colorectal tissues obtained from healthy individuals.

The sample size of the study was calculated using an a priori power analysis performed with G*Power software (version 3.1.9.7). The Wilcoxon signed-rank test was selected for comparing two dependent measurements. An effect size (dz) of 0.53, a significance level (α) of 0.05, and a statistical power (1 − β) of 0.80 were assumed. Based on the analysis conducted for a two-tailed hypothesis test, the minimum required sample size was calculated as 32 participants, with an achieved statistical power of 80.88%.

### 4.2. RNA Isolation

To determine the expression levels of *VEGFA* and *KDR*, colorectal tumor tissues with matched tumor-adjacent non-neoplastic tissue specimens obtained during surgical resection, and cultured colorectal cancer cells were used. Total RNA was isolated from all biological samples using a commercial RNA isolation kit according to the manufacturer’s instructions. Briefly, tissue samples were homogenized in lysis buffer using a tissue homogenizer, whereas cultured cell samples were processed directly according to the manufacturer’s protocol. Total RNA was purified through the recommended binding, washing, and elution procedures.

#### RNA Concentration and Purity Assessment

The concentration and purity of the isolated RNA were evaluated spectrophotometrically using a NanoDrop spectrophotometer (Thermo Fisher Scientific, Waltham, MA, USA). Only RNA samples with an A260/A280 ratio between 1.8 and 2.0 were included in subsequent analyses. RNA samples that met the quality control criteria were stored at −80 °C until cDNA synthesis. To ensure RNA integrity, all procedures were performed under RNase-free conditions.

### 4.3. cDNA Synthesis

Complementary DNA (cDNA) was synthesized from total RNA isolated from colorectal tumor tissues, matched tumor-adjacent non-neoplastic tissues, and cultured colorectal cancer cells according to the manufacturer’s instructions using a commercial cDNA synthesis kit (A.B.T., Berlin, Germany). For each biological sample type, 2 µg of total RNA was used as input for cDNA synthesis. The reverse transcription reaction was performed according to the protocol recommended by the manufacturer. The synthesized cDNA samples were stored at −80 °C until subsequent real-time PCR analyses.

### 4.4. Real-Time PCR Analysis

Real-time PCR analysis was performed using cDNA synthesized from total RNA isolated from colorectal tumor tissues, and matched tumor-adjacent non-neoplastic tissue. Gene expression levels of *VEGFA* (Primer Assay ID: QT01010184) and *KDR* (Primer Assay ID: QT00069818) were determined using commercially available primer assays according to the manufacturer’s instructions. *GAPDH* (Primer Assay ID: PPH00150F) was used as the endogenous reference gene for normalization. All reactions were performed using a commercial real-time PCR master mix under identical experimental conditions, and each sample was analyzed in duplicate. The mean Ct value obtained from the two technical replicates was used for subsequent analyses. Relative gene expression levels were calculated using the 2^−ΔΔCt^ method using the comparative Ct (2^−ΔΔCt^) method.

GAPDH was selected as the endogenous reference gene for normalization of *VEGFA* and *KDR* expression based on its established use in RT-qPCR (Rotor-Gene 6000 Real-Time PCR System QIAGEN, Hilden, Germany) studies. The clinical RT-qPCR analysis included 32 paired colorectal tumor and adjacent non-neoplastic tissue samples, whereas the HT-29 experiments comprised two independent biological replicates (n = 2) per experimental condition. Each biological sample was analyzed in technical duplicate. Relative gene expression levels were calculated using the 2^−ΔΔCt^ method. GAPDH expression stability across the experimental groups and the amplification efficiencies of the target and reference gene assays were not independently validated.

### 4.5. Histopathological Examination

Formalin-fixed, paraffin-embedded (FFPE) colorectal adenocarcinoma tissues and matched tumor-adjacent non-neoplastic colorectal tissues were obtained from 32 patients and retrieved from the archives of the Department of Pathology following approval by the Sivas Cumhuriyet University Health Sciences Research Ethics Committee (approval date: 16 July 2026; approval no: 2026-07/43). Tissue blocks were sectioned at a thickness of 4 μm using a rotary microtome (Leica RM2235, Leica Microsystems, Wetzlar, Germany), and the sections were mounted on poly-L-lysine-coated glass slides. For morphological evaluation, the sections were deparaffinized in xylene, rehydrated through a descending ethanol series, and routinely stained with hematoxylin and eosin (H&E). After staining, the sections were dehydrated through an ascending ethanol series, cleared in xylene, and coverslipped using a permanent mounting medium. Histopathological evaluation was performed in accordance with the fifth edition of the World Health Organization Classification of Tumours of the Digestive System [34]. Sections with well-preserved tissue architecture and minimal technical artifacts were selected for morphological assessment and subsequent immunohistochemical evaluation of VEGFA and VEGFR2 expression.

### 4.6. Immunohistochemistry

Four-micrometer-thick sections obtained from FFPE colorectal adenocarcinoma and matched tumor-adjacent non-neoplastic tissues were deparaffinized in xylene and rehydrated through a descending ethanol series. Heat-induced epitope retrieval was performed in 10 mM citrate buffer (pH 6.0) by heating the sections for 5 min after the buffer reached boiling temperature. The sections were then allowed to cool for 5 min at room temperature. Endogenous peroxidase activity was quenched with 3% hydrogen peroxide for 10 min, and nonspecific antibody binding was blocked with a protein-blocking solution for 60 min at room temperature. Separate sections were incubated overnight at 4 °C with rabbit polyclonal anti-VEGFA antibody (1:100; AF5131, Affbiotech, Cincinnati, OH, USA) or rabbit polyclonal anti-VEGFR2 antibody (1:100; AF6281, Affbiotech, Cincinnati, OH, USA). After three washes with phosphate-buffered saline (PBS), the sections were incubated with an HRP-conjugated anti-rabbit secondary antibody according to the manufacturer’s instructions. Immunoreactivity was visualized using 3,3′-diaminobenzidine (DAB) as the chromogen, and nuclei were counterstained with Mayer’s hematoxylin. The sections were subsequently dehydrated through an ascending ethanol series, cleared in xylene, and coverslipped with a permanent mounting medium. Immunostained sections were examined and photographed using an Olympus BX51 light microscope (Olympus Corporation, Tokyo, Japan). Negative-control sections were processed in parallel under identical conditions, with omission of the primary antibody.

### 4.7. Molecular Docking and ADMET

Molecular docking studies were performed to evaluate the binding affinity of compound **107** against selected colon cancer-related proteins. Protein structures for VEGFA (PDB ID: 3QTK) and VEGFR2 (PDB ID: 2QU5) were obtained from the PDB database [35,36]. Protein and ligand preparations were carried out using AutoDockTools (ADT, version 1.5.6). For the macromolecules, water molecules, co-crystallized ligands, and heteroatoms were removed. Subsequently, polar hydrogens were added, non-polar hydrogen atoms were merged, and partial charges (Gasteiger charges) were computed and added to the protein structures. Compound **107** and the reference ligand DTX were also prepared in ADT, where partial charges were added, non-polar hydrogen atoms were merged, and their torsion tree flexibilities were defined following energy minimization. Docking simulations were performed using AutoDock Vina (version 2021). To encompass the entire active site and allow unrestricted ligand flexibility, the grid box was centered on the native ligand’s binding pocket. A grid box size of 30 × 30 × 30 Å was used for VEGFA (PDB ID: 3QTK) and VEGFR2 (PDB ID: 2QU5), with the grid centers positioned directly over the corresponding native ligand-binding sites. The lowest-energy poses were selected, and ligand–protein interactions were visualized and analyzed using BIOVIA Discovery Studio Visualizer (version 2016) [37]. The lowest-energy pose was selected, and ligand–protein interactions were analyzed using BIOVIA Discovery Studio Visualizer (version 2016) [38]. ADMET properties of compound **107** were predicted using ADMETlab 2.0 [19] to assess its pharmacokinetic, drug-likeness, and toxicity profiles.

### 4.8. Synthesis of Compound ***107***

**Figure 13:**
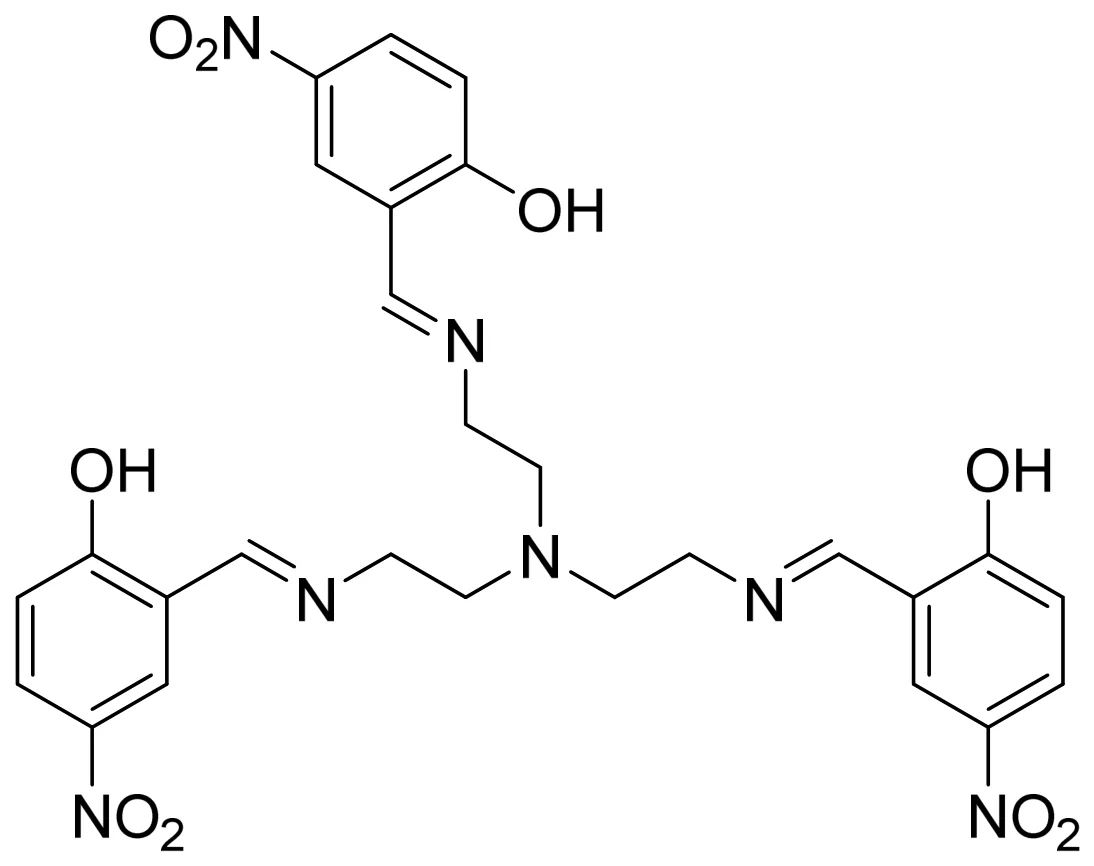
Compound **107** structure.

Compound **107** was synthesized as previously described by Agbektas et al. Briefly, 100 mg of 5-nitrosalicylaldehyde was dissolved in 5 mL of acetonitrile, followed by the addition of 100 µL of tris(2-aminoethyl) amine. The reaction mixture was stirred for 1.5 h under non-catalytic conditions and then poured into ice water to induce precipitation. After approximately 2 h, the resulting yellowish precipitate was collected by filtration, washed with distilled water, and dried at room temperature. The final product was obtained with a yield of 68%, and its structure was confirmed by ^1^H and ^13^C NMR spectroscopy [10] (Figure 13).

### 4.9. Cell Culture Studies

#### 4.9.1. Anticancer Activity Assessment

The human colorectal adenocarcinoma cell line HT-29 (ATCC^®^ HTB-38™) was obtained from the American Type Culture Collection (ATCC, Manassas, VA, USA). HT-29 cells were cultured in RPMI-1640 (RPMI-1640 (Sigma-Aldrich, Taufkirchen, Germany; Cat. No. R8758)) supplemented with 10% fetal bovine serum (FBS; Biological Industries, Beit HaEmek, Israel; Cat. No. 04-007-1A) and 1% penicillin–streptomycin (1%; Gibco™, Thermo Fisher Scientific, Waltham, MA, USA; Cat. No. 15140122) at 37 °C in a humidified incubator containing 5% CO_2_. Cells between passages 10–20 were used for the experiments. Cell morphology and confluence were regularly monitored using an inverted microscope. At approximately 80% confluence, cells were detached using 0.25% trypsin–EDTA (VivaCell Biosciences, Shanghai, China; Cat. No. C3530-0100). For the cell-viability experiments, HT-29 cells were seeded into 96-well plates at a density of 5 × 10^3^ cells/well and allowed to adhere for 24 h before treatment. Compound **107** was dissolved in DMSO, whereas DTX was dissolved in physiological saline, to prepare stock solutions. The stock solutions were subsequently diluted with culture medium to obtain the required treatment concentrations. The final concentration of DMSO in the culture medium did not exceed 0.1% (*v*/*v*). Vehicle-control cells received the same final concentration of the corresponding solvent without Compound **107** or DTX, whereas untreated control cells were maintained under identical culture conditions without drug exposure. To determine the effects of Compound **107** and DTX on HT-29 cell viability and IC_50_ values, cells were treated with different concentrations of Compound **107** and DTX (1, 5, 10, 25, 50, 70, 80, and 100 µg/mL) and incubated for 24, 48, and 72 h. Cell viability was assessed at the end of each incubation period using the MTT assay. Briefly, 10 µL of MTT solution at a concentration of 5 mg/mL was added to each well, and the cells were incubated for 2 h at 37 °C. Following incubation, the medium was removed, and the resulting formazan crystals were dissolved in 100 µL of DMSO. Absorbance was measured at 570 nm using a microplate reader. Each experimental condition was analyzed in three technical replicates, and the experiment was independently repeated three times. Absorbance values were normalized to the corresponding control group, and cell viability was expressed as a percentage (%). IC_50_ values were calculated from the concentration–response data for each incubation period. For consistency with the experimentally applied concentrations, IC_50_ values were expressed in µg/mL. When conversion from µM to µg/mL was required, the values were converted using the molecular weight of each compound according to the following equation: concentration (µg/mL) = concentration (µM) × molecular weight (g/mol)/1000. Based on the determined IC_50_ values, the treatment concentration and incubation period used in subsequent cellular and molecular analyses were selected.

#### 4.9.2. Cell Cycle Analysis

Cell-cycle distribution was determined using the Muse^®^ Cell Cycle Kit (MCH100106; Cytek Biosciences, Fremont, CA, USA) according to the manufacturer’s protocol. Following treatment, approximately 1 × 10^5^–1 × 10^6^ cells were collected for each sample. Cells were centrifuged at 300× *g* for 5 min, washed once with 1 × PBS, and resuspended. While gently mixing the cell suspension, ice-cold 70% ethanol was slowly added to obtain a final cell concentration of approximately 5 × 10^5^–1 × 10^6^ cells/mL. The cells were fixed in 70% ethanol for at least 3 h at −20 °C. Following fixation, 200 µL of the fixed cell suspension was transferred to a new tube, centrifuged at 300× *g* for 5 min, and washed once with 1 × PBS. The cell pellet was then resuspended in 200 µL of Muse^®^ Cell Cycle Reagent and incubated for 30 min at room temperature in the dark. Samples were analyzed using the Guava^®^ Muse^®^ Cell Analyzer (Cytek Biosciences), and DNA-content histograms were used to determine the percentages of cells in the G0/G1, S, and G2/M phases of the cell cycle.

#### 4.9.3. Annexin V Binding Assay

Apoptosis was evaluated using the Muse^®^ Annexin V & Dead Cell Kit (MCH100105; Cytek Biosciences, Fremont, CA, USA) according to the manufacturer’s instructions. Following treatment, both adherent and detached cells were collected to avoid the loss of apoptotic cells. The cells were washed with phosphate-buffered saline (PBS), harvested, and resuspended to obtain a single-cell suspension. Subsequently, 100 µL of Muse^®^ Annexin V & Dead Cell Reagent was added to 100 µL of the cell suspension in each tube. Samples were gently mixed and incubated for 20 min at room temperature in the dark. After incubation, samples were analyzed using the Guava^®^ Muse^®^ Cell Analyzer (Cytek Biosciences). Cells were classified into four populations according to Annexin V and dead-cell marker staining: viable cells (Annexin V−/Dead Cell Marker−), early apoptotic cells (Annexin V+/Dead Cell Marker−), late apoptotic/dead cells (Annexin V+/Dead Cell Marker+), and primarily necrotic/dead cells (Annexin V−/Dead Cell Marker+). Total apoptotic cells were evaluated based on the combined early- and late-apoptotic populations.

#### 4.9.4. Immunofluorescence Staining

Following treatment, HT-29 cells grown on sterile glass coverslips were washed with PBS. The cells were fixed with 4% paraformaldehyde for 15 min at room temperature and washed three times with PBS. Antigen retrieval was subsequently performed in citrate buffer (pH 6.0) by heating the cells for 5 min after the buffer reached boiling temperature, followed by cooling for 5 min at room temperature. Cell membranes were permeabilized with 0.1% Triton X-100 in PBS for 10 min at room temperature, and nonspecific antibody binding was blocked with a protein-blocking solution for 60 min at room temperature in a humidified chamber. Separate coverslips were incubated overnight at 4 °C with rabbit polyclonal anti-VEGFA antibody (1:100; AF5131, Affbiotech, Cincinnati, OH, USA) or rabbit polyclonal anti-VEGFR2 antibody (1:100; AF6281, Affbiotech, Cincinnati, OH, USA). After three washes with PBS for 5 min each, the cells were incubated with an Alexa Fluor 488-conjugated anti-rabbit IgG secondary antibody (1:500; Abcam, Cambridge, UK) for 60 min at room temperature in the dark. The coverslips were washed three times with PBS for 5 min each, and nuclei were counterstained with DAPI for 5 min at room temperature in the dark. After three additional PBS washes, the coverslips were mounted onto glass slides using an antifade mounting medium. Immunofluorescence staining was examined and photographed using an Olympus BX51 fluorescence microscope (Olympus Corporation, Tokyo, Japan) equipped with a × 40 objective. For each marker, all images were acquired using identical illumination intensity, exposure time, gain, and camera settings across the experimental groups. Negative controls were processed in parallel under identical conditions, with omission of the primary antibody. All procedures performed after secondary-antibody incubation were conducted under light-protected conditions.

##### Immunohistochemical Image Analysis

Semi-quantitative analysis of VEGFA and VEGFR2 immunoreactivity was performed using ImageJ version 1.54u8 (National Institutes of Health, Bethesda, MD, USA) [39]. Each immunostained section was first examined in its entirety to evaluate the distribution, intensity, and cellular localization of the immunoreactivity. For quantitative analysis, one microscopic field representing the overall staining pattern of each tissue specimen was selected at ×400 total magnification according to predefined morphological and technical criteria. Eligible fields contained preserved and representative tissue architecture and were free of tissue folds, extensive necrosis, hemorrhage, and staining artifacts. All images within a staining series were acquired using identical illumination, exposure, and camera settings. Images were coded before analysis, and field selection and quantitative measurements were performed by an investigator blinded to tissue identity and clinicopathological information. The DAB chromogenic signal was separated from the hematoxylin counterstain using the Color Deconvolution function with the H DAB vector [40]. The isolated DAB channel was subjected to uniform background correction and converted to optical-density values. Identical threshold and measurement settings were applied to all images analyzed for the same marker. VEGFA and VEGFR2 immunoreactivity was expressed as the integrated optical density (IOD) of the DAB-positive signal within the analyzed field. One IOD value was obtained for each tissue specimen. IOD was interpreted as a semi-quantitative measure of DAB staining under standardized acquisition and analysis conditions, rather than a direct or stoichiometric measure of antigen abundance. Because colorectal adenocarcinoma and tumor-adjacent non-neoplastic tissues were obtained from the same patient, tumor and adjacent-tissue measurements were considered paired biological observations. Thus, the patient, rather than the microscopic field, constituted the experimental unit for the immunohistochemical analysis (n = 32 patients, each contributing one tumor specimen and one matched tumor-adjacent non-neoplastic specimen, yielding 32 paired cases).

### 4.10. Statistical Analysis

Statistical analyses were performed using IBM SPSS Statistics version 23.0 (IBM Corp., Armonk, NY, USA) and GraphPad Prism version 8.0 (GraphPad Software, San Diego, CA, USA). VEGFA and VEGFR2/KDR data were analyzed separately where appropriate. The distribution of continuous variables was assessed using the Shapiro–Wilk test together with graphical inspection. Normally distributed continuous variables were presented as mean ± standard deviation (SD), whereas non-normally distributed variables were summarized as median with interquartile range (IQR). Categorical variables were expressed as number and percentage, n (%). For RT-qPCR analyses of colorectal adenocarcinoma and matched tumor-adjacent non-neoplastic tissues, ΔCt values were calculated by normalizing the Ct value of each target gene to that of the reference gene. Because tumor and matched adjacent tissues were obtained from the same patients, differences in *VEGFA* and *KDR* ΔCt values were evaluated using paired-samples *t*-tests after confirming the normality of the within-patient ΔCt differences. When the normality assumption was not met, the Wilcoxon signed-rank test was used. ΔΔCt was calculated as ΔCt_tumor −ΔCt_matched adjacent tissue, and relative gene expression was determined using the 2^−ΔΔCt^ method. Group-level fold-change values were calculated as 2^−mean ΔΔCt^, with matched adjacent tissue normalized to 1. Differences in tumor-to-adjacent tissue ΔCt values for *VEGFA* and *KDR* among histological grade, tumor localization, and dietary-pattern groups were assessed using the Kruskal–Wallis test. Comparisons according to binary clinical characteristics, including neoadjuvant treatment status, diabetes mellitus, hyperlipidemia, hypertension, cardiovascular disease, history of cancer, alcohol consumption, and smoking status, were performed using the Mann–Whitney U test. Exact two-tailed *p* values were reported when subgroup sizes were small. Associations between continuous demographic variables and tumor-to-adjacent tissue gene-expression differences were evaluated using Spearman’s rank correlation analysis. Spearman’s correlation was also used to assess the relationship between VEGFA and *KDR* tumor-to-adjacent tissue ΔCt differences.

For immunohistochemical analyses, one integrated optical density (IOD) value from the colorectal adenocarcinoma tissue and one IOD value from the matched tumor-adjacent non-neoplastic tissue were available for each of the 32 patients (n = 32 paired observations). Normality of the within-patient differences was evaluated using the Shapiro–Wilk test together with graphical inspection. Tumor and matched adjacent-tissue IOD values were compared using a paired-samples *t*-test when the within-patient differences were normally distributed; otherwise, the Wilcoxon signed-rank test was applied. For HT-29 cell RT-qPCR experiments, statistical comparisons were performed using ΔCt values obtained from independent biological replicates. The effects of treatment and gene type were evaluated using two-way analysis of variance (ANOVA), followed by Dunnett’s multiple-comparisons test to compare the Compound **107**- and DTX-treated groups with the control group within each gene. Relative expression values were calculated using the 2^−ΔΔCt^ method, with the control group normalized to 1, and were used for graphical presentation. For immunofluorescence analyses, the value obtained for each treatment group in each independently performed experiment was considered the unit of statistical analysis (n = 3 biological replicates per group). Differences among the Control, Compound **107**, and DTX groups were analyzed separately for VEGFA and VEGFR2/*KDR* using one-way ANOVA, followed by Tukey’s multiple-comparison test. Individual-cell and field-level measurements were used only to calculate the corresponding biological-replicate value and were not entered into the statistical analyses as independent observations. All statistical tests were two-tailed, and *p* < 0.05 was considered statistically significant. Exploratory subgroup analyses were not adjusted for multiple testing; therefore, these findings were interpreted cautiously, particularly for comparisons involving small or uneven subgroup sizes.

## 5. Conclusions

In conclusion, our study demonstrates increased VEGFA and VEGFR2/*KDR* expression in CRC tissues compared with matched tumor-adjacent non-neoplastic tissues at both the gene and protein levels. The reduced metabolic viability, increased early apoptosis, altered cell cycle distribution, and decreased VEGFA and VEGFR2/*KDR* expression observed in HT-29 cells provide preliminary evidence of the in vitro antitumor effects of Compound **107**. While molecular docking findings support this possibility, they do not prove the direct targeting of VEGFA or VEGFR2. In addition, the present findings do not demonstrate direct VEGFR2 inhibition, VEGFA/VEGFR2 pathway blockade, or direct antiangiogenic activity. Therefore, it is necessary to confirm whether Compound **107** directly affects the VEGFA and VEGFR2/*KDR* pathway through kinase and phosphorylation analyses, functional angiogenesis experiments, and in vivo models, and to evaluate the limitations predicted in ADMET analyses through structural optimization and experimental toxicity studies.

### Limitation

The present study has several limitations that should be considered when interpreting the findings. First, the clinical sample size was relatively small, and all specimens were obtained from a single center. Although available clinicopathological characteristics, including histological grade, tumor location, and neoadjuvant treatment, were evaluated, detailed tumor stage information was unavailable, preventing an assessment of its potential influence on *VEGFA* and *KDR* expression. Furthermore, the comparator group consisted of matched tumor-adjacent histologically non-neoplastic tissues rather than colorectal tissues obtained from healthy individuals. Consequently, the observed expression differences should be interpreted within the context of this paired tissue design. The small and uneven sample sizes in certain clinicopathological subgroups also limited the statistical power and reliability of subgroup analyses.

Second, the in vitro experiments were conducted exclusively using the HT-29 colorectal cancer cell line. Therefore, the findings may not fully represent the molecular heterogeneity of colorectal cancer. Further investigations involving additional colorectal cancer cell lines with distinct molecular characteristics, together with non-malignant colon epithelial cells, are necessary to assess the reproducibility of the observed effects and the potential tumor selectivity of Compound **107**.

Third, although Compound **107** treatment was associated with reduced *VEGFA* and VEGFR2/*KDR* mRNA expression and corresponding protein immunofluorescence signals, these changes cannot be clearly distinguished from secondary effects of reduced cell viability and cellular stress. Concentration–response experiments at sub-cytotoxic doses (IC10–IC25) with matched treatment timepoints were not performed, and unrelated control transcripts were not evaluated to exclude generalized transcriptional alterations. Consequently, the observed molecular changes cannot be considered definitive evidence of selective or direct inhibition of VEGFA/VEGFR2 signaling. Moreover, downstream signaling events, including VEGFR2 phosphorylation, kinase activity, and AKT/ERK pathway activation, were not investigated. The absence of functional angiogenesis assays, such as HUVEC migration and tube formation, further limits conclusions regarding the potential antiangiogenic activity of Compound **107**. Future studies incorporating sub-cytotoxic treatment conditions, additional control transcripts, and functional pathway analyses are required to clarify whether the observed changes reflect pathway-specific regulation or secondary consequences of cytotoxicity.

Fourth, the RT-qPCR analyses have methodological limitations. Although commercially available primer assays were used and GAPDH was selected as the endogenous reference gene based on its established use in gene expression studies, its expression stability was not independently validated across paired colorectal tumor and adjacent non-neoplastic tissues or different HT-29 treatment conditions. In addition, the amplification efficiencies of the VEGFA, *KDR,* and GAPDH assays were not experimentally determined, and no-template and no-reverse-transcription negative controls were not included. Therefore, the assumption of comparable amplification efficiencies underlying the 2^−ΔΔCt^ method could not be verified. These limitations may introduce uncertainty into the relative gene expression measurements and warrant cautious interpretation of the molecular findings. Future investigations should incorporate standard-curve-based amplification efficiency assessments, appropriate negative controls, and systematic reference gene stability validation to improve the accuracy and reproducibility of RT-qPCR results.

Additionally, mycoplasma screening was not performed during the study; therefore, the possibility of mycoplasma contamination and its potential influence on cellular responses cannot be completely excluded.

Finally, the anticancer efficacy and potential systemic toxicity of Compound **107** have not been evaluated in in vivo models, including colorectal cancer xenografts. Furthermore, the ADMET findings were derived exclusively from computational predictions and therefore do not provide experimental confirmation of pharmacokinetic behavior or toxicological safety. Additional in vivo studies, together with experimental pharmacokinetic and toxicological assessments, are necessary to establish the biological relevance, safety profile, and therapeutic potential of Compound **107**.

## Figures and Tables

**Table 1:** Demographic, clinical, and clinicopathological characteristics of the patients.

Variable	Patients (n = 32)
Age, years, mean ± SD	64.78 ± 9.73
Gender, n (%)	
Male	21 (65.6)
Female	11 (34.4)
Alcohol consumption, n (%)	
No	31 (96.9)
Yes	1 (3.1)
Smoking status, n (%)	
No	24 (75.0)
Yes	8 (25.0)
Hyperlipidemia, n (%)	
No	30 (93.8)
Yes	2 (6.3)
Cardiovascular disease, n (%)	
No	27 (84.4)
Yes	5 (15.6)
History of cancer, n (%)	
No	21 (65.6)
Yes	11 (34.4)
Tumor localization, n (%)	
Right colon	3 (9.4)
Hepatic flexure	2 (6.3)
Transverse colon	1 (3.1)
Splenic flexure	3 (9.4)
Descending colon	3 (9.4)
Sigmoid colon	1 (3.1)
Rectosigmoid colon	12 (37.5)
Upper rectum	3 (9.4)
Middle rectum	3 (9.4)
Lower rectum	1 (3.1)
Neoadjuvant therapy, n (%)	
No	30 (93.8)
Yes	2 (6.3)
Histological grade, n (%)	
Grade 1	9 (28.1)
Grade 2	21 (65.6)
Grade 3	2 (6.3)

Data are presented as mean ± standard deviation (SD) for age and as number and percentage, n (%), for categorical variables.

**Table 2:** Comparison of *VEGFA* and *KDR* expression between tumor and matched tumor-adjacent non-neoplastic tissues.

Gene	Tumor Tissue ΔCt, Mean ± SD	Matched Tumor-Adjacent Non-Neoplastic Tissues ΔCt, Mean ± SD	Mean ΔΔCt (95% CI)	*t*	*p*	Fold Change (2^−mean ΔΔCt^)
VEGFA	5.80 ± 2.88	6.78 ± 2.75	−0.98 (−1.14 to −0.83)	−13.21	<0.001 *	1.98
KDR	11.30 ± 2.48	12.31± 2.30	−1.01 (−1.20 to −0.82)	−10.84	<0.001 *	2.02

ΔCt values are presented as mean ± standard deviation (SD). Tumor and matched tumor-adjacent non-neoplastic tissues were compared using the paired-samples *t*-test. ΔΔCt was calculated as ΔCt_tumor − ΔCt_matched tumor-adjacent non-neoplastic tissue. Fold change was calculated as 2^−mean ΔΔCt^. A fold-change value greater than 1 indicates higher gene expression in tumor tissue. * Statistically significant at *p* < 0.05.

**Table 3:** Comparison of *VEGFA* and *KDR* ΔΔCt values according to histological grade.

Gene	Grade 1, Median (Min–Max)	Grade 2, Median (Min–Max)	Grade 3, Median (Min–Max)	Kruskal–Wallis *H*	*p*-Value
*VEGFA* ΔΔCt	−1.05 (−1.44 to −0.60)	−1.07 (−1.68 to −0.13)	−1.28 (−1.66 to −0.90)	0.760	0.684
*KDR* ΔΔCt	−0.65 (−1.54 to −0.55)	−0.95 (−1.92 to −0.24)	−1.85 (−2.35 to −1.34)	3.130	0.209

Data are presented as median (minimum–maximum). Differences among histological grade groups were assessed using the Kruskal–Wallis test. Grade 1, Grade 2, and Grade 3 included 9, 21, and 2 patients, respectively. Findings for Grade 3 should be interpreted cautiously because of the small sample size. Statistically significant at *p* < 0.05.

**Table 4:** Correlation between *VEGFA* and *KDR* tumor-to-adjacent-tissue ΔCt differences.

Variable 1	Variable 2	Spearman’s ρ	*p* Value	N
*VEGFA* tumor-to-adjacent-tissue ΔCt difference	*KDR* tumor-to-adjacent-tissue ΔCt difference	0.358	0.044 *	32

Correlation was assessed using Spearman’s rank correlation analysis. * Statistically significant at *p* < 0.05.

**Table 5:** Compound 107’s physicochemical, lipophilicity and ADMET parameters.

Property	107	Comment
Molecular Weight	593.19	Molecular Weight < 500
nHA	16	Hydrogen bond acceptors < 12
nHD	3	Hydrogen bond donors < 7
logP	2.82	Log of the octanol/water partition coefficient: 0–5
TPSA	230.43	Topological Polar Surface Area: 0–140
HIA	0.00	Human Intestinal Absorption Category 1: HIA+ (HIA ≥ 30%); Category 0: HIA− (HIA ≤ 30%); The output value is the probability of being HIA+.
Caco-2 Permeability	−5.387	Optimal: higher than −5.15 Log units
BBB	0.00	Blood–Brain Barrier Penetration The output value is the probability of being BBB+
PPB	82.026	Plasma Protein Binding Optimal: <90%. Drugs with high protein binding may have a low therapeutic index.
VD	0.057	Volume Distribution Optimal: 0.04–20 L/kg
CYP2D6	0.033	Category 1: Inhibitor; Category 0: Non-inhibitor; The output value is the probability of being an inhibitor.
CYP3A4	0.013	Category 1: Inhibitor; Category 0: Non-inhibitor; The output value is the probability of being an inhibitor.
T_1/2_	1.081	The unit of predicted T_1/2_ is hours. ultra-short half-life drugs: 1/2 < 1 h; short half-life drugs: T_1/2_ between 1–4 h; intermediate short half-life drugs: T_1/2_ between 4–8 h; long half-life drugs: T1/2 > 8 h.
hERG Blockers	0.796	Category 1: active; Category 0: inactive; The output value is the probability of being active.
H-HT	0.81	Human Hepatotoxicity Category 1: H-HT positive (+); Category 0: H-HT negative (−); The output value is the probability of being toxic.
AMES Toxicity	0.999	Category 1: Ames positive (+); Category 0: Ames negative (−); The output value is the probability of being toxic.
Rat Oral Acute Toxicity	0.794	Category 0: low-toxicity; Category 1: high-toxicity; The output value is the probability of being highly toxic.

**Table 6:** Docking scores and RMSD values against PDB ID: 3QTK and PDB ID: 2QU5 for 107 and DTX compounds.

Compounds	PDB: 3QTK		PDB: 2QU5	
	Docking Score (kcal/mol)	RMSD (A)	Docking Score (kcal/mol)	RMSD (A)
107	−8.60	1.50	−8.30	1.70
DTX	−8.30	1.60	−8.00	1.80

**Table 7:** The interaction parameters between **107**, DTX Compounds and the 3QTK enzyme.

Type of Bond	Interacting Amino Acid (107)	Bond Length (Å)	Interacting Amino Acid (DTX)	Bond Length (Å)
Van der Waals Interactions	LYS-41 LEU-59 CYS-53 GLU-60	-	CYS-44 LEU-59 GLY-52 LYS-100	-
Conventional H-Bonds	TYR-14 GLU-57 SER-43 ASN-55 CYS-54 ARG-98	2.81 2.88 2.52 3.43 3.33, 3.37 2.92, 3.10	ARG-98 CYS-54 ASN-55	2.81 2.77 2.96
Carbon Hydrogen Bond	ASP-27	3.84	ASP-27 SER-43	3.66 3.41
Unfavorable Donor–Donor	LYS-101	4.93	-	-
Pi–Cation	LYS-100 ASP-56	5.74 5.16	-	-
Pi–Anion	GLU-57	5.08	GLU-57	4.67
Pi–Alkyl	PRO-99 ILE-36 ILE-39	5.41 6.64 6.30	ILE-39 - -	6.32 - -
Pi–Sigma	-	-	PRO-99	5.58
Pi–Pi T-Shaped	-	-	PHE-29	5.68
Pi–Pi Stacked	-	-	PHE-29	4.86

**Table 8:** The interaction parameters between **107**, DTX Compounds and the 2QU5 enzyme.

Type of Bond	Interacting Amino Acid (107)	Bond Length (Å)	Interacting Amino Acid (DTX)	Bond Length (Å)
Van der Waals Interactions	SER-1100 ARG-932 LYS-1110 TYR-1106	-	ALA-881 ILE-1111 MET-1072 ALA-1050	-
Conventional H-Bonds	ASP-1129 ILE-1111 ARG-929 SER-925 SER-1104 GLU-1102	3.28 3.02 3.16 2.90 2.58, 3.54 3.42	ARG-1066 LEU-1049 ARG-1080 TYR-1082	3.47 3.18, 3.41 3.48 2.76
Carbon Hydrogen Bond	PRO-1128	3.52	-	-
Pi–Cation	LYS-1070	5.28	ARG-1066	5.45
Pi–donor Hydrogen Bond	SER-1104	4.32	-	-
Pi–Sigma	ALA-1103	4.23	-	-
Pi–Alkyl	LEU-1067	6.75	LEU-1069 PRO-1068 ALA-844	4.52 5.40 6.06
Pi–Anion	-	-	ASP-1064	5.94
Alkyl	-	-	LEU-1049	4.75

**Table 9:** IC_50_ values of Compound 107 and DTX in HT-29 cells following different incubation periods. IC_50_: half-maximal inhibitory concentration; DTX: docetaxel.

	HT-29 Cell Line		
	IC_50_ (µg/mL ± SD *)		
Incubation Period	24 h	48 h	72 h
Compound 107	32.91 ± 0.14	19.96 ± 0.05	17.51 ± 1.36
DTX	80.79 ± 1,22	61.26 ± 0.02	37.86 ± 1.54

IC_50_: half-maximal inhibitory concentration. SD *: Standard Deviation.

## Data Availability

The original contributions presented in this study are included in the article. Further inquiries can be directed to the corresponding author.

## Abbreviations

The following abbreviations are used in this manuscript:

ADMET	Absorption, Distribution, Metabolism, Excretion, and Toxicity
AKT	Protein Kinase B
ANOVA	Analysis of Variance
BBB	Blood–Brain Barrier
CRC	Colorectal Cancer
CTCF	Corrected Total Cell Fluorescence
DAB	3,3′-Diaminobenzidine
DAPI	4′,6-Diamidino-2-phenylindole
DTX	Docetaxel
ESM1	Endothelial Cell-Specific Molecule 1
FBS	Fetal Bovine Serum
FFPE	Formalin-Fixed, Paraffin-Embedded
H&E	Hematoxylin and Eosin
HIA	Human Intestinal Absorption
HIF-1α	Hypoxia-Inducible Factor-1 Alpha
HSP60	Heat Shock Protein 60
HUVEC	Human Umbilical Vein Endothelial Cell
IC_50_	Half-Maximal Inhibitory Concentration
IHC	Immunohistochemistry
IOD	Integrated Optical Density
KDR	Kinase Insert Domain Receptor
LINC	Long Intergenic Non-Protein Coding RNA
MAPK	Mitogen-Activated Protein Kinase
miRNA	MicroRNA
MTT	3-(4,5-Dimethylthiazol-2-yl)-2,5-Diphenyltetrazolium Bromide
NMR	Nuclear Magnetic Resonance
PBS	Phosphate-Buffered Saline
PDB	Protein Data Bank
PI	Propidium Iodide
PI3K	Phosphoinositide 3-Kinase
PPB	Plasma Protein Binding
PTEN	Phosphatase and Tensin Homolog
qRT-PCR	Quantitative Reverse Transcription Polymerase Chain Reaction
RNA	Ribonucleic Acid
SD	Standard Deviation
TPSA	Topological Polar Surface Area
Treg	Regulatory T Cell
USP13	Ubiquitin-Specific Peptidase 13
VD	Volume of Distribution
VEGF	Vascular Endothelial Growth Factor
VEGFA	Vascular Endothelial Growth Factor A
VEGFR2	Vascular Endothelial Growth Factor Receptor 2
Wnt	Wingless/Integrated
YTHDF1	YTH N6-Methyladenosine RNA-Binding Protein 1

## References

[1] Yang J., Li C., Wang Z., Jiang K. (2024). Multi-omics analysis of the biological function of the VEGF family in colon adenocarcinoma. Funct. Integr. Genom..

[2] Zheng D., Zhang X., Xu J., Chen S., Wang B., Yuan X. (2024). LncRNA LINC01503 promotes angiogenesis in colorectal cancer by regulating VEGFA expression via miR-342-3p and HSP60 binding. J. Biomed. Res..

[3] Tsumuraya H., Mimura K., Nakajima S., Hanayama H., Matsuishi A., Okayama H., Fukai S., Ito M., Ashizawa M., Chida S. (2024). Tumor infiltrating effector regulatory T cells express VEGF receptor 2 in patients with colorectal cancer. Anticancer Res..

[4] Xie Z., Niu L., Du K., Chen L., Zheng G., Dai S., Dan H., Duan L., Dou X., Feng F. (2024). Endothelial cell heterogeneity in colorectal cancer: Tip cells drive angiogenesis. Cell Mol. Life Sci..

[5] Ma R.T., Wang Y., Ji F., Chen J.N., Wang T.J., Liu Y., Hou M.X., Guo Z.G. (2024). YTHDF1’s grip on CRC vasculature: Insights into LINC01106 and miR-449b-5p-VEGFA axis. Cancer Cell Int..

[6] Xu G.Z., Guan H.Y., Guo Y.G., Zhang Y.R., Pan J.H., Luo S., Ding H., Pan Y., Yao Q. (2025). USP13 suppresses colorectal cancer angiogenesis by downregulating VEGFA expression through inhibition of the PTEN-AKT pathway. Oncol. Res..

[7] Haroon M., Kang S.C. (2025). Kaempferol promotes apoptosis and inhibits proliferation and migration by suppressing HIF-1α/VEGF and Wnt/β-catenin activation under hypoxic condition in colon cancer. Appl. Biol. Chem..

[8] Corrias G., Lai E., Ziranu P., Mariani S., Donisi C., Liscia N., Saba G., Pretta A., Persano M., Fanni D. (2024). Prediction of response to anti-angiogenic treatment for advanced colorectal cancer patients: From biological factors to functional imaging. Cancers.

[9] Jacobsen A., Siebler J., Grützmann R., Stürzl M., Naschberger E. (2024). Blood vessel-targeted therapy in colorectal cancer: Current strategies and future perspectives. Cancers.

[10] Agbektas T., Zontul C., Ozturk A., Huseynzada A., Ganbarova R., Hasanova U., Cinar G., Tas A., Kaya S., Chtita S. (2023). Effect of azomethine group containing compounds on gene profiles in Wnt and MAPK signal patterns in lung cancer cell line: *İn silico* and *in vitro* analyses. J. Mol. Struct..

[11] Shaldam M.A., Abdulla M.-H., Angeli A., Hefny S.M., El-Labbad E.M., Obeed A.B., Alhassan N.S., Supuran C.T., Eldehna W.M., Tawfik H.O. (2024). Novel sulfonamide-tethered Schiff bases as anti-proliferative agents with VEGFR-2 inhibitory activity: Synthesis, biological assessment, and molecular dynamic simulations. J. Mol. Struct..

[12] Li T., Tian Y., Wang Y., Yang J., Chen Z., Li Y. (2025). Elucidation of novel diagnostic biomarkers and therapeutic targets in colorectal carcinoma: An integrative approach leveraging multi-omics, computational biology, and single-cell sequencing technologies. Mamm. Genome.

[13] Parra-Sánchez Á., Román-Vallina A., Fernández E., Martínez-Navarrete G. (2026). Computational modeling of anti-VEGFA drug interactions with VEGF-A: Insights into therapeutic strategies for neovascular AMD. Comput. Biol. Chem..

[14] Mohanty B., Munshi P. (2025). Disulfide-rich polysulfur alkaloids target vascular endothelial growth factor A through electronic duality and shape-responsive recognition. Sci. Rep..

[15] Hu L., Xu Y., Yao K., Wang X., Xu J., Li Y., Hu S. (2026). Structure-based design, synthesis, and biological evaluation of benzofuran derivatives as dual TGF-βR1 and VEGFR2 inhibitors for potent antitumor therapy. Bioorg. Chem..

[16] Abdelsalam Ouf A.M., Marzouk A.A., Shaykoon M.S.A., Celik I., Bakhotmah D.A., Mansour A.A., Alamoudi M.K., Alfayomy A.M., El-Zahabi M.A. (2025). Synthesis, antiproliferative evaluation and in silico studies of new quinazoline derivatives as VEGFR-2 inhibitors. Future Med. Chem..

[17] Vardhan S., Sahoo S.K. (2020). *İn silico* ADMET and molecular docking study on searching potential inhibitors from limonoids and triterpenoids for COVID-19. Comput. Biol. Med..

[18] Yalçın S. (2020). Molecular docking, drug likeness, and ADMET analyses of Passiflora compounds as P-glycoprotein (P-gp) inhibitor for the treatment of cancer. Curr. Pharmacol. Rep..

[19] ADMETlab2.0. A Platform for Systematic ADMET Evaluation. https://admetmesh.scbdd.com/.

[20] Yang Z., Zhang X., Bai X., Xi X., Liu W., Zhong W. (2024). Anti-angiogenesis in colorectal cancer therapy. Cancer Sci..

[21] Long Y., Dan Y., Jiang Y., Ma J., Zhou T., Fang L., Wang Z. (2024). Colorectal cancer cell-derived extracellular vesicles promote angiogenesis through JAK/STAT3/VEGFA signaling. Biology.

[22] Prager G.W., Taieb J., Fakih M., Ciardiello F., Van Cutsem E., Elez E., Cruz F.M., Wyrwicz L., Stroyakovskiy D., Pápai Z. (2023). Trifluridine-tipiracil and bevacizumab in refractory metastatic colorectal cancer. N. Engl. J. Med..

[23] Liu C., Meng L., Wang L., Dong B., Qu L., Zhao C., Shou C. (2026). Gamma-synuclein drives bevacizumab resistance in colorectal cancer via VEGFR2 activation and angiogenesis. Drug Resist. Updates.

[24] Seliem I.A., Panda S.S., Girgis A.S., Tran Q.L., Said M.F., Bekheit M.S., Abdelnaser A., Nasr S., Fayad W., Soliman A.A.F. (2022). Development of isatin-based Schiff bases targeting VEGFR-2 inhibition: Synthesis, characterization, antiproliferative properties, and QSAR studies. Chem. Med. Chem..

[25] Abd El-Lateef H.M., Elbastawesy M.A.I., Ibrahim T.M.A., Khalaf M.M., Gouda M., Wahba M.G.F., Zaki I., Morcoss M.M. (2023). Design, synthesis, docking study, and antiproliferative evaluation of novel Schiff base-benzimidazole hybrids with VEGFR-2 inhibitory activity. Molecules.

[26] Elkady H., El-Dardir O.A., Elwan A., Taghour M.S., Mahdy H.A., Dahab M.A., Elkaeed E.B., Alsfouk B.A., Ibrahim I.M., Husein D.Z. (2023). Synthesis, biological evaluation and computer-aided discovery of new thiazolidine-2,4-dione derivatives as potential antitumor VEGFR-2 inhibitors. RSC Adv..

[27] Başaran E., Çakmak R., Türkmenoğlu B., Akkoc S., Köprü S. (2025). Synthesis of sulfonamide-based Schiff bases as potent anticancer agents: Spectral analyses, biological activity, molecular docking, ADME, DFT, and pharmacophore modelling studies. Chem. Biodivers..

[28] Sahin Dogan S., Cicek M., Aydin D., Kocabas A. (2025). Evaluation of the therapeutic potential of a Schiff base compound: Pharmacokinetic profiling, molecular docking for enoyl-acyl carrier protein reductase (InhA), antimicrobial, and anticancer properties. Int. J. Biol. Macromol..

[29] Karaosmanoğlu O., Berber H., Sivas H., Uysal U.D. (2025). Exploring Schiff bases derived from 2-hydroxybenzaldehyde as poten-tial anticancer agents: Synthesis, characterization, molecular docking and in-vitro evaluation. Cell Biochem. Biophys..

[30] Postigo-Corrales F., Beltrán-Videla A., Lázaro-Sánchez A.D., Hurtado A.M., Conesa-Zamora P., Arroyo A.B., Luengo-Gil G. (2025). Docetaxel resistance in breast cancer: Current insights and future directions. Int. J. Mol. Sci..

[31] Moshawih S., Lim A.F., Ardianto C., Goh K.W., Kifli N., Goh H.P., Jarrar Q., Ming L.C. (2022). Target-based small molecule drug discovery for colorectal cancer: A review of molecular pathways and in silico studies. Biomolecules.

[32] Aziz M.A., Serya R.A.T., Lasheen D.S., Abdel-Aziz A.K., Esmat A., Mansour A.M., Singab A.N.B., Abouzid K.A.M. (2016). Discovery of potent VEGFR-2 inhibitors based on furopyrimidine and thienopyrimidine scaffolds as cancer targeting agents. Sci. Rep..

[33] Taştemur Ş., Kaleci A.O., Öztürk A., Mendil A.S. (2025). Astaxanthin promotes apoptosis by suppressing growth signaling pathways in HT-29 colorectal cancer cells. Med. Oncol..

[34] Nagtegaal I.D., Odze R.D., Klimstra D., Paradis V., Rugge M., Schirmacher P., Washington K.M., Carneiro F., Cree I.A. (2020). WHO Classification of Tumours Editorial Board. The 2019 WHO classification of tumours of the digestive system. Histopathology.

[35] R. P. D. Bank (2011). The Crystal Structure of Chemically Synthesized VEGF-A. https://www.rcsb.org/structure/3QTK.

[36] R. P. D. Bank (2007). Crystal Structure of the VEGFR2 Kinase Domain in Complex with a Benzimidazole Inhibitor. https://www.rcsb.org/structure/2QU5.

[37] Eberhardt J., Santos-Martins D., Tillack A.F., Forli S. (2021). AutoDock Vina 1.2.0: New docking methods, expanded force field, and Python bindings. J. Chem. Inf. Model..

[38] BIOVIA Discovery Studio (2016). Discovery Studio Visualizer.

[39] Schneider C.A., Rasband W.S., Eliceiri K.W. (2012). NIH Image to ImageJ: 25 years of image analysis. Nat. Methods.

[40] Ruifrok A.C., Johnston D.A. (2001). Quantification of histochemical staining by color deconvolution. Anal. Quant. Cytol. Histol..

